# Syncytiotrophoblast of Placentae from Women with Zika Virus Infection Has Altered Tight Junction Protein Expression and Increased Paracellular Permeability

**DOI:** 10.3390/cells8101174

**Published:** 2019-09-29

**Authors:** Jael Miranda, Dolores Martín-Tapia, Yolotzin Valdespino-Vázquez, Lourdes Alarcón, Aurora Espejel-Nuñez, Mario Guzmán-Huerta, José Esteban Muñoz-Medina, Mineko Shibayama, Bibiana Chávez-Munguía, Guadalupe Estrada-Gutiérrez, Samuel Lievano, Juan Ernesto Ludert, Lorenza González-Mariscal

**Affiliations:** 1 Department of Physiology, Biophysics and Neuroscience, Center for Research and Advanced Studies (Cinvestav), Mexico City 07360, Mexico; jamiguz_316@hotmail.com (J.M.); dolores@fisio.cinvestav.mx (D.M.-T.); lalarcon@fisio.cinvestav.mx (L.A.); 2 Research Division, Instituto Nacional de Perinatología (INPer) Isidro Espinosa de los Reyes, Mexico City 11000, Mexico; yolotzin@gmail.com (Y.V.-V.); aurora_espnu@yahoo.com.mx (A.E.-N.); mguzmanhuerta@yahoo.com.mx (M.G.-H.); gpestrad@gmail.com (G.E.-G.); 3 Laboratorio Central de Epidemiología, Instituto Mexicano del Seguro Social, Ciudad de México 02990, Mexico; jose.munozm@imss.gob.mx; 4 Department of Infectomics and Molecular Pathogenesis, Center for Research and Advanced Studies (Cinvestav), Mexico City 07360, Mexico; mineko@cinvestav.mx (M.S.); bchavez@cinvestav.mx (B.C.-M.); jludert@cinvestav.mx (J.E.L.); 5 Quality division, Obstetrics and Gynecology Hospital No. 4, Mexican Institute of Social Security (IMSS), Mexico City 01090, Mexico; samuel.lievano@yahoo.com.mx

**Keywords:** zika virus, flavivirus, tight junctions, claudins, ZO-1, blood-placental barrier, placenta

## Abstract

The cytotrophoblast of human placenta transitions into an outer multinucleated syncytiotrophoblast (STB) layer that covers chorionic villi which are in contact with maternal blood in the intervillous space. During pregnancy, the Zika virus (ZIKV) poses a serious prenatal threat. STB cells are resistant to ZIKV infections, yet placental cells within the mesenchyme of chorionic villi are targets of ZIKV infection. We seek to determine whether ZIKV can open the paracellular pathway of STB cells. This route is regulated by tight junctions (TJs) which are present in the uppermost portion of the lateral membranes of STB cells. We analyzed the paracellular permeability and expression of E-cadherin, occludin, JAMs –B and –C, claudins -1, -3, -4, -5 and -7, and ZO-1, and ZO-2 in the STB of placentae from ZIKV-infected and non-infected women. In ZIKV-infected placentae, the pattern of expression of TJ proteins was preserved, but the amount of claudin-4 diminished. Placentae from ZIKV-infected women were permeable to ruthenium red, and had chorionic villi with a higher mean diameter and Hofbauer hyperplasia. Finally, ZIKV added to the basolateral surface of a trophoblast cell line reduced the transepithelial electrical resistance. These results suggest that ZIKV can open the paracellular pathway of STB cells.

## 1. Introduction

The placental syncytiotrophoblast (STB) layer that covers the floating chorionic villi in direct contact with maternal blood in the intervillous space constitutes a major barrier to vertical transmission of parasites and microorganisms. The consequences of STB damage have been studied in first trimester placental explants where the subsyncytial cytotrophoblasts (CTB) becomes infected by *Listeria monocytogenes* if the STB layer is damaged with collagenase [1]. With *Toxoplasma gondii*, the situation is more complex, as multiparasite vacuoles in chorionic villi are present not only immediately below or besides syncytial interruptions, but also at sites of no visible syncytial interruptions, suggesting the possibility of hard to detect breaks in the STB layer or of earlier breaks that had subsequently healed [2]. These observations highlight the importance of considering syncytial permeability upon studying placental vulnerability to infection.

Several viruses cross the uterine-placental interface, infecting the fetus and causing birth defects, including rubella, varicella-zoster, parvovirus B19, human cytomegalovirus, hepatitis E type 1 and Zika (ZIKV) (see [3]). ZIKV is a flavivirus which is transmitted by *Aedes* mosquitoes; it was first identified in 1947 in an African forest, and triggered epidemics in the South Pacific in 2007 and in the Americas in 2015–2017 [4]. ZIKV infection during pregnancy is associated to an array of devastating birth defects known as congenital Zika syndrome, which include microcephaly, brain calcifications, neurological impairment, and retinal damage.

We are interested in exploring the mechanisms that allow ZIKV transplacental transmission in humans. During the development of human placenta, CTB epithelial cells may be differentiated in two ways. Firstly, they aggregate into cellular columns that invade the uterine interstitium and colonize the spiral arterioles, allowing the anchorage of the fetus to the mother and the flow of blood to the placenta. Secondly, CTB form a bilayer in which the cells of the external sheet fuse to form the multinucleated STB that covers the chorionic villi. The STB layer is crucial for the interchange of ions, nutrients, gases, and waste between the fetus and the mother. During pregnancy, as a result of syncytial ruptures or focal degeneration of STB, lateral cell membranes subdivide segments of STB from the surrounding STB continuum. This appears to be a dynamic process where the disconnected parts of the STB eventually fuse after the disintegration of the lateral separating membranes [5].

Tight junctions (TJs) regulate transit through the paracellular pathway of epithelial cells. In the STB, these cell-cell adhesion structures located at the uppermost portion of the lateral membranes that subdivide the STB layer constitute a cornerstone of the blood-placental barrier (BPB) that protects the fetus from toxins and pathogens. TJs in the STB of human placental chorionic villi have been observed by freeze fracture, and their function as paracellular seals has been demonstrated by electron microscopy in thin sections, with the blockade of the transit of electron-dense markers through the paracellular pathway [6,7,8,9]. The apical surface of the STB of chorionic villi expresses several TJ proteins, including the integral proteins occludin, claudins -1, -3 and -16, and the adaptor protein ZO-1 [9,10], while claudin-4 is strongly expressed during all trimesters of pregnancy [11], but localizes at the basal membrane of the STB [9].

ZIKV infects cells that strongly express TIM-1 [12], a cell surface phosphatidylserine and phosphatidylethanolamine receptor [13]. ZIKV in humans replicates in the glandular epithelium of the decidua and in decidual cells and infects invasive CTB, the CTB of cell columns, as well as fetal fibroblasts and macrophages known as Hofbauer cells which are present in the parenchyma of floating chorionic villi [12,14,15]. These observations led to the proposal of a ZIKV transmission route that goes from the cells of the basal decidua in the mother to the fetal invading CTB, followed by the infection of cell columns of CTB and Hofbauer cells present in the parenchyma of chorionic villi [12]. Moreover, since the envelope (E) proteins between the dengue virus (DENV) and ZIKV are very similar structurally [16], cross-reactive antibodies are generated that may enhance ZIKV infection [17]. In this respect, it has been observed that ZIKV infection in human placental explants are enhanced by the presence of dengue virus antibodies, suggesting that ZIKV immune complexes could use the neonatal Fc receptor for IgG as a transport system to transcytose across the STB layer to infect Hofbauer cells in the chorionic villi [18]. However, since the clinical severity of maternal ZIKV infection has not being associated with the existence of prior dengue antibodies [19], and not all pregnant women infected with ZIKV have been previously infected with DENV, here, we explore another complementary route of vertical ZIKV transmission. Taking into account that STB cells are not infected by ZIKV [12], we explore the possibility of ZIKV reaching the chorionic mesenchyme via transit through the paracellular route of the STB.

Virus passage through the paracellular route of epithelial and endothelial cells has previously been reported. Thus, after human airway epithelia infection with adenovirus, the viruses are first released to the basolateral surface and then escape to the apical surface. This process involves the binding of the fiber proteins of the adenovirus to its receptor CAR located within TJs, which triggers a disruption of junctional integrity that allows the virus to escape through the paracellular pathway between the cells to reach the apical surface [20]. Another case is that of human immunodeficiency virus (HIV), whose disruption of TJs and adherens junctions (AJs) in oral epithelial cells facilitates the paracellular spread of the herpes simplex virus 1 (HSV1), emerging as a mechanism to explain the rapid development of HSV-associated oral lesions in HIV infected individuals [21]. Likewise, sealing the blood-brain barrier (BBB) is compromised by HIV-1 induced inflammatory cytokines; in this respect, TNF-α has been shown to open the paracellular route for HIV-1 invasion across the BBB [22]. With regards to *Flaviviruses*, the West Nile virus disrupts the BBB in mice inducing an increase in BBB permeability and a reduced expression of TJ and AJ proteins [23]; moreover, in vitro infection of mouse brain endothelia with DENV delocalizes TJ proteins from the membrane to the cytoplasm, reduces the transendothelial electrical resistance, and increases the macromolecule permeability and the paracellular passing of free virus particles [24].

Here, we analyzed the permeability of the paracellular pathway and the molecular composition of TJs in the STB cell layer of placentae derived from ZIKV-infected women. In addition, in the trophoblast cell line BeWo, we observed that basolateral incubation with ZIKV reduces the transepithelial electrical resistance (TER).

Our results indicate that ZIKV infection alters the composition of placental TJs and increases paracellular permeability.

## 2. Materials and Methods

### 2.1. Ethics Statement

All subjects gave their informed consent for inclusion before they participated in this study. This study was approved by the Ethics Committee of the Instituto Nacional de Perinatologia (INPer) Isidro Espinosa de los Reyes in Mexico City (Register 212250-1000-10107-01-16) and San Judas Tadeo Hospital in Mexico City.

### 2.2. Patient Selection and Specimens

Placental tissues were obtained from 4 ZIKV-infected women and 4 control women. In the control women, the absence of ZIKV, Chikungunya virus (CHIKV), and DENV was confirmed by RT-PCR, done in placental tissue at the Central Epidemiological Laboratory of the Mexican Institute of Social Security (IMSS). For the 4 ZIKV-infected women, the RT-PCR diagnosis of ZIKV was made during the acute phase of ZIKV infection. In the newborns of the 4 ZIKV-infected women, detection of ZIKV was confirmed by RT-PCR on neonatal serum, amniotic fluid, and umbilical cord by the Mexican Institute of Diagnosis and Epidemiological Reference (InDRE), and the absence of CHIKV, DENV, and West Nile virus in these samples was confirmed by RT-PCR done by the National Institute of Respiratory Diseases (INER).

The pregnancies of ZIKV-infected women enrolled in this study were closely followed at INPer in Mexico City. A multidisciplinary clinical team, including maternal-fetal clinicians, geneticists, and pediatric neurologists, assessed the ultrasonographic diagnoses of described fetal alterations; prenatal findings were confirmed at birth. Immediately after giving birth by cesarean section, placental samples were fixed and processed as described below.

### 2.3. Cell Culture

BeWo cells were obtained from the American Type culture Collection (ATCC^®^ CCL-98™) and cultured at 37 °C and 95% air 5% CO_2_ in Ham′s F12K medium (Cat. N3520, SIGMA-ALDRICH, St. Louis, MO, USA), supplemented with 10% fetal calf serum (Cat. 26140-079, Gibco, Grand Island, NY, USA) and 1% penicillin/streptomycin (Cat. 150770, Invitro, S.A., CDMX, Mexico). Cells were plated on Transwell filters (12-well plate, 0.4 µm, Cat. 3460, Corning, Kennebunk, ME, USA); 24 h later, the monolayers were incubated for 1.5 h with ZIKV at MOI = 1. Then, the monolayers were fixed for 10 min with methanol at −20 °C for claudin-4 immunofluorescence, or fixed in 4% para-formaldehyde in PBS at RT for 10 min and permeabilized with 0.2% Triton X-100 in PBS for occludin and ZO-1 immunofluorescence.

### 2.4. Immunofluorescence

Immunofluorescence on placental cryostate sections was done as previously reported [25], with the exception that the incubation with the first antibody was done overnight (ON) at 4 °C; the procedure of tissue fixation varied according to the protein to be detected as follows: (1) for E-cadherin, claudins -2 and -7, occludin, and ZO-1, the tissue was fixed in acetone at −20 °C for 5 min; (2) for claudins -3 and -4, and JAMs –B and –C, the samples were fixed in ethanol at 4 °C for 10 min followed by fixation in acetone at −20 °C for 3 min; (3) for ZO-2 and claudins -1 and -5, the tissue was fixed in 4% para-formaldehyde in PBS at RT for 10 min. As primary antibodies, a mouse monoclonal against cytokeratin 18 was employed (Cat. MAB1600, Chemicon International, Temecula, CA, USA; dilution 1:5000), together with one of the following rabbit polyclonal antibodies: anti E-cadherin (Cat. 3195, Cell Signaling Technology, Inc., Danvers, MA, USA; 1:300); anti claudin-1 (Cat. 51–9000, Invitrogen, Camarillo, CA, USA; dilution 1:100); anti claudin-3 (Cat. Ab52231, abcam, Cambridge, MA, USA; dilution 1:100); anti claudin-4 (Cat. 36–4800, Invitrogen, Camarillo, CA, USA; dilution 1:300); anti claudin-5 (Cat. ab15106, Abcam, San Diego, CA, USA; dilution 1:300); anti claudin-7 (Cat. 34–9100, Invitrogen, Camarillo, CA; dilution 1:100); anti occludin (Cat. 71–1500, Invitrogen, Carlsbad, CA, USA; dilution 1:100); anti ZO-1 (Cat. 61–7300, Invitrogen, Carlsbad, CA; dilution 1:300); and anti ZO-2 (Cat. 71–1400, Invitrogen, Carlsbad, CA; dilution 1:100). We also employed mouse monoclonal antibodies anti claudin-2 (Cat. sc-293233, Santa Cruz Biotechnology, Santa Cruz, CA, USA; dilution 1:100); and (1) anti JAM-B (Cat. sc-293496, Santa Cruz Biotechnology, Santa Cruz, CA; dilution 1:100); and a rat monoclonal antibody anti JAM-C (Cat. MCA5935, CRAM18F26, SeroTec, Kidlington, UK; dilution 1:100). As secondary antibodies, we employed a donkey antibody against mouse IgG coupled to Alexa594 (Cat. A21203, Invitrogen, Carlsbad, CA; dilution 1:1000), a donkey antibody against rabbit IgG coupled to Alexa-488 (Cat. 21206, Invitrogen, Carlsbad, CA; dilution 1:1000), and donkey antibody against goat IgG coupled to Alexa-488 (Cat. A21208, Invitrogen, Carlsbad, CA; dilution 1:1000).

### 2.5. Relative Mean Fluorescence Intensity Measurements

Relative mean fluorescence intensity measurements of AJ and TJ proteins at the STB were obtained using ImageJ (ImageJ 1.52n, NIH, Bethesda, MD, USA, 2019) with the Freehand function. An area named A, surrounding the chorionic villi, was selected. Then, another area named B, of the parenchyma of the same villi, immediately below the region of the STB, was selected. The integrated density feature of ImageJ was used to record pixel intensities of each of these two areas. Then, the integrated density of area A minus that of area B was recorded and compared to the fluorescent signal of the STB. Data were derived from three randomly-selected fields per placenta, and the images shown in the figures were one of the quantitated fields.

Data were derived from three images per placenta.

### 2.6. Western Blot

Western blots of placental lysates were done following standard procedures, as previously described [25]. As primary antibodies, we employed the following: rabbit polyclonal antibodies anti E-cadherin (Cat. 3195, Cell Signaling Technology, Inc., Danvers, MA; 1:3000); anti claudin-1 (Cat. 51–9000, Invitrogen, Camarillo, CA; dilution 1:1000); anti claudin-2 (Cat. 51–6100, Invitrogen, Camarillo, CA; dilution 1:1000); anti claudin-3 (Cat. Ab52231, Abcam, Cambridge, MA; dilution 1:500); anti claudin-4 (Cat. 36–4800, Invitrogen, Camarillo, CA; dilution 1:4000); anti claudin-5 (Cat. ab15106, Abcam, San Diego, CA; dilution 1:2000); anti claudin-7 (Cat. 34–9100, Invitrogen, Camarillo, CA; dilution 1:500); anti occludin (Cat. 71–1500, Invitrogen, Carlsbad, CA; dilution 1:1000); anti ZO-1 (Cat. 61–7300, Invitrogen, Carlsbad, CA; dilution 1:1000); and anti ZO-2 (Cat. 71–1400, Invitrogen, Carlsbad, CA; dilution 1:500). We also employed mouse monoclonal antibodies anti JAM-C (Cat. sc-515893, Santa Cruz Biotechnology, Santa Cruz, CA; dilution 1:500) and anti-JAM-B (Cat. sc-293496, Santa Cruz Biotechnology, Santa Cruz, CA; dilution 1:500). As secondary antibodies, we employed goat antibodies against rabbit IgG coupled to peroxidase (Cat. 6111620, Invitrogen, Camarillo, CA; dilution 1:20,000), or against mouse IgG coupled to peroxidase (Cat. 626520, Invitrogen, Camarillo, CA; dilution 1:10,000).

### 2.7. Histochemical Staining

Chorionic villi derived from the placentae of control and ZIKV-infected women were fixed in 10% para-formaldehyde, embedded in paraffin, and cut in 8 μm sections. Then sections were stained with hematoxylin and eosin, or with Masson’s trichrome stain, following standard protocols [26]. The identification of Hofbauer cells in these slides was done with a rat monoclonal antibody against CD68 (Cat. ab53444, Abcam, San Diego, CA; dilution 1:1,000).

### 2.8. Transmission Electron Microscopy (TEM)

The placental tissues of control and ZIKV-infected women were fixed in 2.5% glutaraldehyde in 0.1 M sodium cacodylate buffer for 1 h at room temperature (RT). Then, the tissue was incubated for 20 h at 4 °C in 2.5% glutaraldehyde in 0.1 M sodium cacodylate buffer, washed thrice with 0.1 M sodium cacodylate buffer, and incubated for 1 h at RT with 1% osmium tetroxide in 0.1 M sodium cacodylate buffer containing 0.5 mg/mL of ruthenium red, a marker of the paracellular pathway. Samples were then processed for TEM, as previously described [27].

### 2.9. Measurement of Transepithelial Electrical Resistance (TER)

The transepithelial electrical resistance of BeWo cells plated at confluence on Transwell filters (12-well plate, 0.4 µm, Cat. 3460, Corning, Kennebunk, ME) was continuously measured from each insert using the automated cell monitoring system, cellZscope (nanoAnalytics GmbH, Munster, Germany). TER values were obtained using the cellZscope software version 4.3.1. Twenty-four hours after plating, when the monolayers had achieved a steady state value of TER, cells were infected with ZIKV (strain PRVABC-59-Asian, ATCC^®^ VR-1843™) at a MOI of 1.

### 2.10. Statistical Analysis

The data derived from control and ZIKV-infected placentae obtained from the relative mean fluorescence intensity images and Western blot densitometry analysis were compared for statistically significant differences using the Mann-Whitney *U* test. The variances obtained with claudin-4 results were equal among the two groups; therefore, we applied a Student’s *t*-test to include effect size estimations; since both groups had similar standard deviations, we used Cohen’s *d*. For the Western blots of occludin, the data were further analyzed to include effect size estimation with Glass’delta, since each group had a different standard deviation. The Graphs were generated with GraphPad Prism 5.01 software (GraphPad Prism 5.01, La Jolla, CA, USA, 2007).

## 3. Results

### 3.1. The Trophoblast of Chorionic Villi in Human Placentae Expressed E-cadherin and a Wide Array of TJ Proteins

In this study, we analyzed the expression of TJ proteins in the placentae from control and ZIKV-infected women. The clinical data and perinatal outcomes of control and ZIKV-infected women during pregnancy and their newborns are summarized in Table 1 and Table 2. With regards to the newborn of ZIKV-infected donor D8, that carries a 22q11 deletion, all the reported phenotypic characteristic of the child conform with 22q11 deletion syndrome.

In the trophoblast of chorionic villi in placentae derived from control and ZIKV-infected women during pregnancy, we explored the expression of the AJ protein E-cadherin and of the following TJ proteins: claudins -1, -2, -3, -4, -5, -7, and -10; JAMs -A, -B, and -C; occludin, ZO-1, and ZO-2 (Table 3). All these proteins, with the exception of claudins -2, -10, and JAM-A (data not shown), were expressed in the STB of chorionic villi.

### 3.2. E-cadherin Stained the Basal Membrane of the STB and Its Expression was not Affected in ZIKV-Infected Placentae

In chorionic villi of human placentae, E-cadherin was observed not at the apical surface of the STB layer in contact with the intervillous space, which in situ is occupied by maternal blood, but in the basal membrane of the STB in contact with remnants of the CTB layer and the chorionic mesenchyma (Figure S1a). In this and most of the subsequent images, the trophoblast layer was identified with cytokeratin 18, a canonical marker of epithelial cells [28]. The intensity of the fluorescent signal of E-cadherin in the STB layer was not affected in placentae derived from ZIKV-infected women (Figure S1b). Likewise, by Western blot, no difference in the amount of E-cadherin was observed in placental lysates from control and ZIKV-infected women (Figure S1c,d).

### 3.3. The Expression of Claudin-1 Slightly Increased in Placentae Derived from ZIKV-Infected Women

By immunofluorescence, we observed that claudin-1 stained fetal endothelia within the chorionic villi parenchyma as well as the STB layer. The relative mean fluorescence intensity of the signal in the STB layer slightly increased in placentae from ZIKV-infected women in comparison to control placentae, albeit not at a significant level (Figure S2a,b). By Western blot, we observed that the amount of claudin-1 was not significantly higher in placentae derived from ZIKV-infected women in comparison to control placentae (Figure S2c,d).

### 3.4. The Expression of Claudin-3 was Strong in Placental Vessels and Faint at the STB Layer of Both Control and ZIKV-Infected Placentae

*Claudin-3* was strongly expressed in the vessels of the parenchyma in chorionic villi, while staining at the STB cell layer was weak in both control and ZIKV-infected placentae (Figure S3a,b). By Western blot, we detected no difference in the amount of claudin-3 between control and ZIKV-infected placentae (Figure S3c,d).

### 3.5. The Expression of Claudin-4 at the Basolateral Membrane of STB Diminished in ZIKV-Infected Placentae

**Figure 1:**
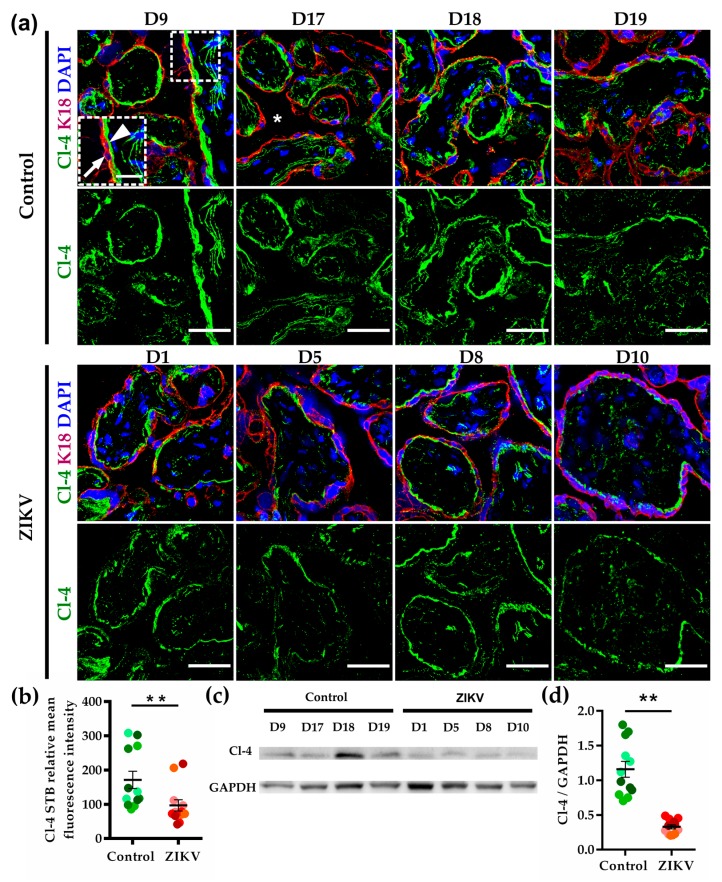
Claudin-4 present at the basal surface of the STB layer diminished in ZIKV-infected placentae. (**a**) Frozen sections of human placentae derived from women infected or not (control) with ZIKV were processed for immunofluorescence with a rabbit antibody against claudin-4 (Cl-4) and a mouse antibody anti cytokeratin 18 (K18). DNA of nuclei was stained with DAPI. Apical surface of STB cell layer (arrow); basolateral surface of STB cell layer (arrowhead); intervillous space (asterisk). Bar, 50 μm; magnification bar, 25 μm. (**b**) Measurements of mean fluorescence intensity of the trophoblast layer were done on three independent images from each condition. Since the variances were equal among the two groups, we applied a Student’s *t*-test. To include effect size estimation, since both groups have similar standard deviations, we used Cohen’s *d*. The three values obtained per donor are represented by dots with the same color. ** *p* = 0.0054, Cohen’s *d* = 3 indicating that 99.9% of the values from the ZIKV group are below the mean value of the control group. (**c**) Representative Western blot of three independent experiments. Glyceraldehyde 3-phosphate dehydrogenase (GAPDH) was employed as loading control. (**d**) Densitometric analysis of Western blots. The three values obtained per donor are represented by dots with the same color. ** *p* = 0.00092 Cohen’s *d* = 2.67 indicating that 99.9% of the values from the ZIKV group are below the mean value of the control group.

In the chorionic villi of control human placentae, claudin-4 was observed at the basolateral membrane of the STB in contact with the chorionic parenchyma. The same pattern was observed in the placentae from ZIKV-infected women; however, in this case, the fluorescent signal at the STB layer was less intense (Figure 1a,b). Western blot analysis confirmed the decreased expression of claudin-4 in ZIKV-infected placentae in comparison to those of the control (Figure 1c,d).

### 3.6. Claudin-5 Strong Expression in the Vessels of Chorionic Villi and Faint Stain in STB Cells Did not Change with ZIKV Infection

Claudin-5 was previously identified as an endothelial claudin [29], and accordingly, strongly stained the vessels within the chorionic parenchyma. In addition, claudin-5 faintly stained the STB cell layer in both ZIKV-infected and in control placentae (Figure S4a,b). Western blot analysis also revealed that ZIKV infection induced no change in the amount of claudin-5 expressed in the placentae (Figure S4c,d). The Western blot was done with a placental lysate, and hence, contains claudin-5 from both the vessels and the STB. In the case of claudin-5, this situation is particularly relevant, as this claudin is more abundant in vessels than in the STB cell layer.

### 3.7. Claudin-7 Expression at the Basal Surface of STB Cells was not Altered by ZIKV Infection

In the chorionic villi of control and ZIKV-infected placentae, claudin-7 was preferentially expressed in the basal membrane of STB, although a faint staining was also detected at the apical surface (Figure S5a,b). Immunofluorescence quantitation of claudin-7 at the STB cell layer and Western blot analysis showed no change in claudin-7 abundance in ZIKV-infected placentae (Figure S5c,d).

### 3.8. Occludin Expression in the STB was Low and not Affected by ZIKV Infection

**Figure 2:**
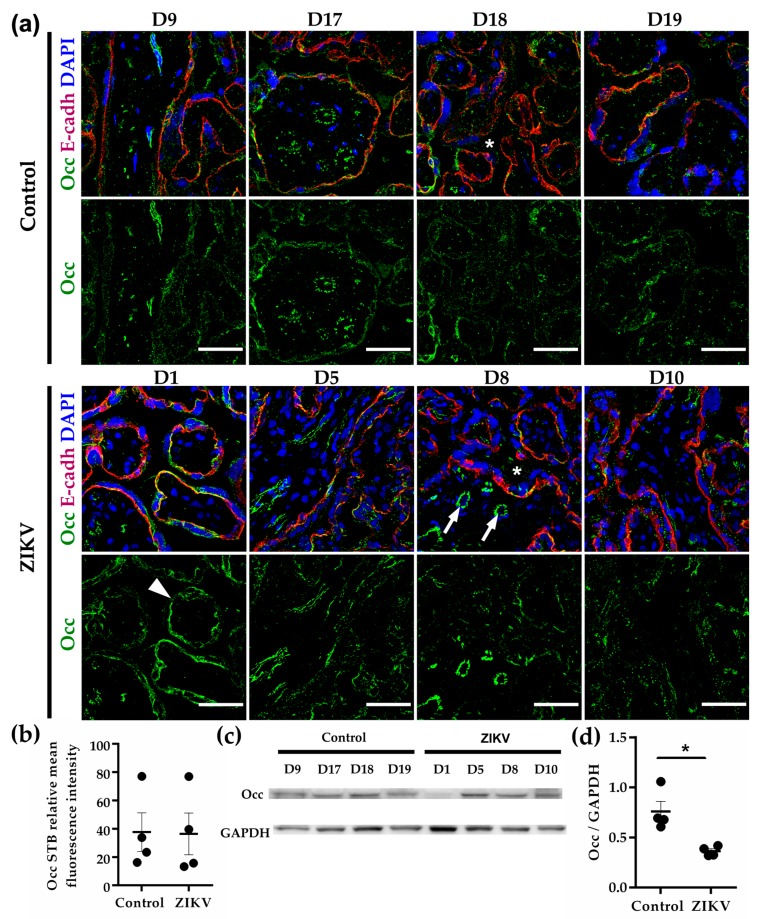
Occludin expression at chorionic vessels is stronger than in the cell layer. (**a**) Frozen sections of human placentae derived from women infected or not (control) with ZIKV were processed for immunofluorescence with a mouse antibody against occludin (Occ) and a rabbit antibody anti E-cadherin (E-cadh). DNA of nuclei were stained with DAPI. Chorionic vessels (arrows); STB (arrowhead); intervillous space (asterisks). Bar, 50 µm. (**b**) Measurements of mean fluorescence intensity of trophoblast layer were done on three independent images from each condition. (**c**) Representative Western blot of three independent experiments. GAPDH was employed as loading control. (**d**) Densitometric analysis of Western blots. * *p* = 0.0286. The data were further analyzed to include effect size estimation with Glass’delta since each group had a different standard deviation. The value of Glass’delta = 1.955 indicates that 99.9% of the values from the ZIKV group are below the mean value of the control group.

In chorionic villi, occludin was strongly expressed in parenchymal vessels and faintly stained the STB cell layer in both control and ZIKV-infected placentae (Figure 2a,b). Western blot analysis showed a decrease in occludin expression in response to ZIKV infection (Figure 2c,d). These Western blots, however, most likely reflect the amount of occludin present in endothelial TJs rather than in the STB cell layer.

### 3.9. The Expression of ZO-1 was Higher in Chorionic Vessels than in the STB and was not Affected by ZIKV Infection

ZO-1 strongly stained the vessels in the chorionic villi and delineated the STB layer in a moderate manner, although in some placentae from both control (D19) and ZIKV-infected women (D10), labeling at the STB was more intense (Figure S6a,b). Western blot analysis revealed a similar amount of ZO-1 in both group of placentae, and most likely reflects the content of ZO-1 at endothelia, due to the higher expression of this protein in vessels in comparison to the STB (Figure S6c,d).

### 3.10. In Chorionic Villi, the Expression of ZO-2 in the STB was Much Higher than in the Mesenchymal Vessels

*ZO-2* in the chorionic villi of human placentae was clearly expressed in the STB, while it was barely present in the vessels of the mesenchyme. By immunofluorescence, only in one ZIKV-infected placentae (D8), the amount of ZO-2 increased in comparison to the rest of the placentae (Figure S7a,b) and the Western blot analysis showed no change in ZO-2 expression induced by ZIKV infection (Figure S7c,d).

### 3.11. ZIKV Infection Had no Impact on the Expression of JAMs -B and -C in the STB of Chorionic Villi

JAM-B stained with a similar intensity the STB cell layer of both control and ZIKV-infected placentae. Dots of JAM-B were also present in the chorionic mesenchyme, but not along the TJs of chorionic vessels, which were conspicuously stained with ZO-1 (Figure S8a,b). By Western blot, similar amounts of JAM-B were found between control and ZIKV-infected placentae (Figure S8c,d). In contrast, JAM-C was not observed at the STB cell layer and profusely stained the endothelial vessels in the chorionic mesenchyme. In the control and ZIKV-infected placentae, the same JAM-C staining pattern was observed (Figure S9a,b), and no change in the amount of JAM-C was detected by Western blot after ZIKV infection (Figure S9c,d).

In summary, the exploration of E-cadherin and a wide variety of TJ proteins revealed that E-cadherin and claudins -4 and -7 were present in the basal membrane of the STB, while all the other TJ proteins studied localized in both the apical and basal membranes of the STB. The pattern of expression of these proteins was preserved in ZIKV-infected placentae, although in this pathological condition, the amount of claudin-4 diminished in the STB.

### 3.12. The STB Layer of Placentae of ZIKV-Infected Women is Permeable to Ruthenium Red

**Figure 3:**
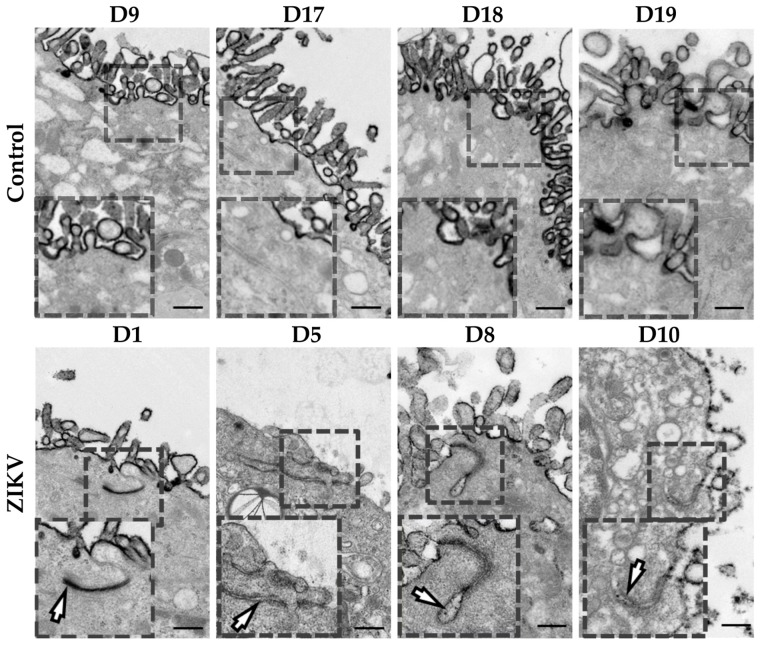
The STB in the placentae of ZIKV-infected women was permeable to ruthenium red. Placental tissue was fixed and processed for TEM in the presence of ruthenium red. Ruthenium red staining in the paracellular pathway (arrows). Bar, 1 μm.

Since claudin-4 functions as a cationic barrier [30] or an anion pore [31] that increases TER in cationic and anion selective cell lines and decreases paracellular permeability in the cationic selective cell line MDCK [32], we next determined whether the TJs of ZIKV-infected placentae were leaky. For this purpose, placental tissue was fixed and processed for TEM in the presence of ruthenium red, an electron-dense paracellular marker. In all the ZIKV-infected placentae, we observed ruthenium red staining in the paracellular pathway bellow the TJ region in the STB cell layer, in contrast to control placentae, where, as we had previously shown [9], staining was restricted to the apical membrane of the STB (Figure 3).

### 3.13. Hofbauer Cell Hyperplasia, an Increased Diameter of Microvilli and Intravillous Calcifications were Observed in ZIKV-Infected Placentae

**Figure 4:**
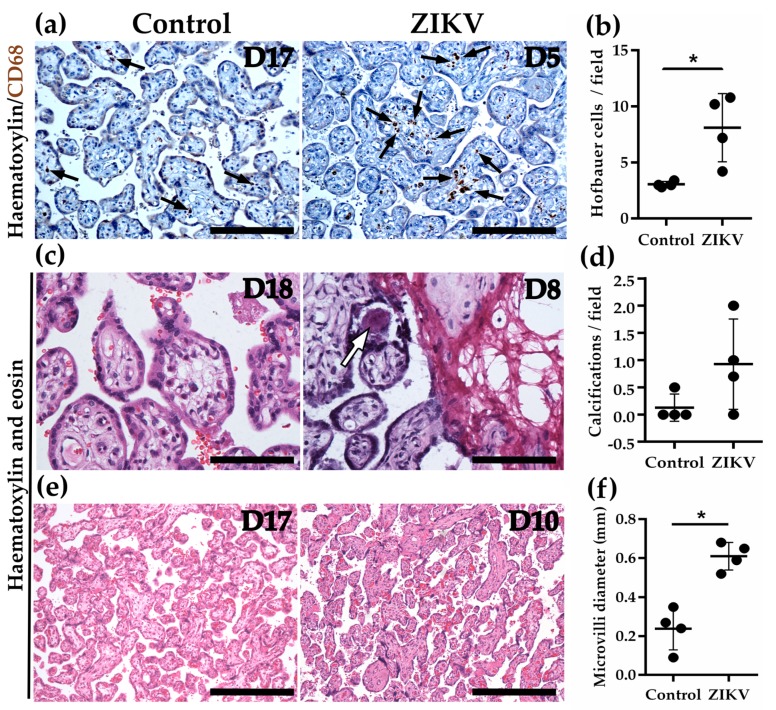
The chorionic villi of ZIKV-infected placentae display Hofbauer cell hyperplasia and a higher mean diameter. (**a**) Hofbauer cells (arrows) in chorionic villi were detected with an antibody against CD68 in slides stained with haematoxylin. Bar, 100 µm. (**b**) The number of Hofbauer cells was evaluated counting CD68+ positive cells in five optical fields per placenta. Each dot corresponds to the mean value of Hofbauer cells/field present in each placenta. * *p* = 0.0294. (**c**) Calcification (arrow) present in a chorionic villus detected in a slide stained with haematoxylin and eosin. Bar, 50 µm. (**d**) Calcifications were counted in five optical fields per placenta. Each dot corresponds to the mean value of calcifications/field present in each placenta. (**e**) The diameter of chorionic microvilli is higher in ZIKV-infected placentae than in the control condition. Bar, 200 µm. (**f**) The diameter of chorionic villi was measured using the image analysis software Zen (version ZEN 2.3 lite, Carl Zeiss Microscopy, Jena, Germany) in five optical fields of samples from placentae stained with haematoxylin and eosin. Each dot corresponds to the mean diameter of chorionic villi per placenta. * *p* = 0.0286.

The chorionic villi derived from women infected with ZIKV during pregnancy displayed several histological alterations, including: (1) Hofbauer cell hyperplasia, evaluated by counting the number of CD68+ cells in the parenchyma of floating chorionic villi stained with hematoxylin (Figure 4a,b); (2) intravillous calcifications observed in hematoxylin and eosin-stained samples, which show a tendency to increase in ZIKV placentae in comparison to control, but whose difference is not statistically significant (Figure 4c,d); and (3) a higher diameter of chorionic microvilli in hematoxylin and eosin-stained samples (Figure 4e,f).

Some placentae infected with ZIKV also displayed chorionic villi edema, heterogeneous maturation of chorionic villi, characterized by the co-existence of villi with different diameters; increased syncytial knots due to premature aging; and karyorrhexis, the irregular distribution of chromatin in the cytoplasm due to the destructive fragmentation of the nucleus of dying cells (Figures S10–S13). In contrast, tissue sections of chorionic villi from the placentae of control women displayed a homogeneous maturation of chorionic villi, an absence of inflammatory cells, and a parenchyma without abundant Hofbauer cells (Figures S14–S17).

We also performed a Masson’s trichrome stain in the chorionic villi of D1 placenta from a ZIKV positive woman in order to detect pathological changes involving the connective tissue. Supplemental Figure S18 reveals perivascular fibrosis and abundant intravilli collagen, as well as mesenchymal edema and karyorrhexis, which were previously observed with the hematoxylin and eosin stain.

### 3.14. ZIKV Added to the Basolateral Surface of the Trophoblast-Derived Cell Line BeWo Reduces the Transepithelial Electrical Resistance and Claudin-4 Expression

**Figure 5:**
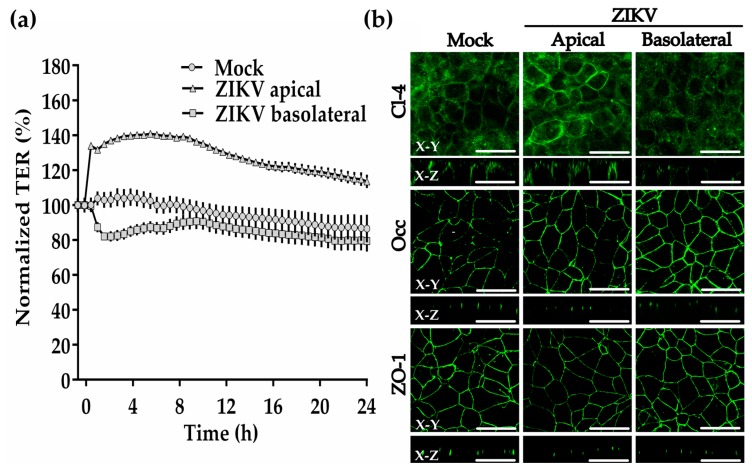
ZIKV added to the basolateral surface of BeWo cells transiently diminishes the transepithelial electrical resistance and claudin-4 expression. Confluent monolayers of BeWo cells that had achieved a stable value of TER were incubated with ZIKV (MOI = 1) added to the apical or basolateral surface. (**a**) TER was continuously measured in the cellZscope system in three inserts per condition. Results are shown with the corresponding standard deviation. (**b**) Immunofluorescence for claudin-4, occludin and ZO-1 in BeWo monolayers done after 1.5 h of incubation with ZIKV. Bar, 50 μm. X-Y, en face view; X-Z, lateral view.

To further explore the effect of ZIKV on TJs of the trophoblasts, we incubated ZIKV at a MOI of 1 with the trophoblast-derived choriocarcinoma cell line BeWo cultured on Transwell filters. We worked with BeWo cells because they constitute a human cell culture model of placental villous trophoblast, and because the STB layer of human placenta is poorly susceptible to infection by ZIKV [33]. Figure 5a shows that the apical administration of ZIKV immediately increases TER by 34%, reaches 40% above control after 6 h, and diminishes to 27% above control after 24 h. Instead, the administration of ZIKV to the basolateral surface of BeWo cells induced a fast drop of TER of 20% in comparison to control monolayers at 1.5 h. However, after 10 h, the values of TER had recovered and were undistinguishable from those of control monolayers. Hence, these results reveal that ZIKV in contact with the basolateral surface of trophoblast cells is able to reduce the degree of sealing of TJs.

By immunofluorescence, we observed no change in the expression of occludin or ZO-1 in BeWo cells, 1.5 h after the addition of ZIKV to the apical or basolateral surfaces (Figure 5b). Instead, the expression of claudin-4 diminishes 1.5 h after ZIKV is added to the basolateral surface. In addition, while occludin and ZO-1 concentrate as spots at the TJ region in immunofluorescence z-sections, claudin-4 distributes along the basolateral membrane, as had been previously observed in intestinal Caco-2 cells [34] These results thus confirm the capacity of ZIKV to diminish the expression of claudin-4 in trophoblasts.

## 4. Discussion

TJs in the STB are a cornerstone of the BPB that protects the fetus from toxins and pathogen infections. Given the devastating consequences of ZIKV fetal infection, in this work, we explored the expression of E-cadherin and TJ proteins in the STB of human placentae of women infected with ZIKV. By immunofluorescence, we observed that E-cadherin, claudins -1, -3, -4, -5, and -7; JAM-B; occludin, ZO-1, and ZO-2 were present in the STB of chorionic villi from both control and ZIKV-infected women. Only JAM-A and claudins -2 and -10 (data not shown) were not detected in these tissues. In contrast, in mice, a strong induction of claudin-10 was observed in the decidua at pregnancy day 4.5, although in humans, claudin-10 was not detected in first trimester decidual cells [35].

Claudins -1, -3, and -5, JAM-B, occludin, ZO-1, and ZO-2 are present in both the apical and basal surfaces of the STB layer, whereas E-cadherin and claudins -4 and -7 concentrate at the basal surface of the STB in contact with the underlying CTB or chorionic parenchyma. This pattern of expression was not altered by ZIKV infection.

The TJ proteins that were more conspicuously expressed in the STB layer were claudins -1, -3, -4, -7, JAM-B, occluding, and ZO-2. These results agree with previous observations showing strong expression of claudin-4 in trophoblastic cells during all trimesters of human pregnancy [11] and with our previous results with occludin and claudins -1, -3, and -4 in the placentae of control women and with preeclampsia [9]. In addition, in mice placentae, a real-time PCR study revealed a higher level of expression of mRNA for claudins -1, -2, -4, and -5 in comparison to all other claudins in the family, while a Western blot revealed an increased expression of claudin-4 and -5 and a decrease in the content of claudin-2 as pregnancy advances from day 12 to 20 [36].

Here, we observed that both occludin and ZO-1 delineated the STB layer in a moderate manner in comparison to the strong staining observed in the chorionic vessels. Therefore, the decreased content of occludin detected by Western blot in the placentae from ZIKV-infected women is most likely due to a reduction in the amount of occludin present in endothelia, particularly since the immunofluorescence signal of occludin at the STB layer did not decrease in the placentae from ZIKV-infected women. Therefore, we think that the diminished expression of occludin in the endothelia of the chorionic parenchyma does not contribute to the leakiness observed in the STB layer of chorionic villi in placentae derived from ZIKV-infected women.

These observations, however, do not minimize the importance of ZO-1 and occludin for a healthy STB, particularly since in hydatidiform moles, characterized by hyperplasia of the trophoblastic tissue and distention of the chorioninc villi by fluid, the expression of ZO-1 and occludin is downregulated and their distribution in the STB changes from the cell borders to the cytoplasm [10]. In addition, ZO-1 appears to play a crucial role in the fusion of trophoblastic cells into a syncytium, as ZO-1 expression at intercellular boundaries decreases during fusion; the treatment of primary human trophoblastic cells in culture with ZO-1 siRNA blocks this process [37].

The expression of occludin in fetal vessels of human placenta is observed mainly at term [10], and occurs in the secondary chorionic villi in large and intermediate vessels but not in terminal exchange vessels, as we and others have shown [9,38]. Instead, the expression of *ZO-1* in fetal endothelia has been observed throughout gestation [10] and among the whole placental vascular tree [38]. In this respect, it is interesting to note that one of the reasons that ZO-1 knock out mice are embryonic lethal is due to abnormal angiogenesis in the yolk sac [39].

With regards to ZO-2, it is interesting to note that in contrast to *ZO-1*, this protein was abundantly expressed in the STB layer of human placentae but barely detectable in chorionic vessels. Unlike ZO-1 knock out mice, ZO-2 knock out mice do not die due to alterations in angiogenesis [39]. Instead, ZO-2 knock out mice are embryonic lethal due to defects in the development of the extraembryonic tissue. This was demonstrated when the injection of ZO-2^−/−^ embryonic stem cells into wild type blastocysts generated viable ZO-2 chimera mice [40].

Our results indicate a reduced expression of claudin-4 in BeWo cells when ZIKV was added to the basolateral surface and in the STB layer of chorionic villi from ZIKV-infected women. The change observed in the chorionic villi cannot be attributed to the preeclampsia present in two of the ZIKV-infected donors, because the placentae of ZIKV-infected women without preeclampsia also exhibited a reduced claudin-4 expression. In addition, a histochemistry study reported a slight increase in *claudin-4* expression in the trophoblasts of preeclamptic placentae [11]. Finally, by Western blot, we previously observed that the amount of claudin-4 in the chorionic villi does not vary with preeclampsia [9].

The differences in the gestational age between the ZIKV-infected placentae and the controls arose because women infected with ZIKV were subjected to earlier cesarean sections due to their high-risk pregnancies in order to better protect fetal health, while healthy women in the control group had cesarean sections at the expected time for full-term pregnancies. Nevertheless, the differences in claudin expression here observed cannot be ascribed to the variation in gestational age between the groups, because in humans, there are no differences in the level of expression of claudin-4 in trophoblastic cells of chorionic villi, between the three trimesters of pregnancy [11].

The combination and mixing ratios of claudin species determines the barrier properties of TJ strands [41], and the alteration of a single type of claudin can significantly alter the permeability and transepithelial electrical resistance of a tissue [30,32]. Therefore, the decrease in claudin-4 expression in placentae from ZIKV-infected woman may have a big impact in the paracellular transit through the STB, particularly since this claudin functions as a cation barrier [30,32] or an anion pore [31]. Accordingly, a significant decrease in permeability and an increase in TER have been observed in MDCK cells after claudin-4 transfection [30,32].

Claudin-4 in the renal collecting duct interacts with claudin-8, and their association is required to form a paracellular chloride channel [31]. Therefore, it may be important in the future to determine whether claudin-8 is expressed together with claudin-4 in the STB layer in human placentae, and whether its expression is altered in ZIKV-infected placentae.

Claudin-4 is a critical claudin for the establishment of a permeability barrier to protect the developing embryo. When the blastocyst enters the uterus, the process of implantation and placentation starts. The first contact is established between the blastocyst trophoectoderm and the uterine epithelium. Once the blastocyst attaches, the process of decidualization is triggered, involving the stromal epithelial transition in which uterine stromal cells differentiate into decidual cells surrounding the implanting blastocyst. In this event, the trophoectoderm acts as a stimulus for the creation of a TJ permeability barrier in stromal cells that protects the embryo from the passage of injurious maternal immunoglobulins [42,43]. In rat stromal cells of the uterus, we have observed that claudin-1 is present in all gestational days, and that ZO-1 appears until day 6, albeit at both implantation and non-implantation sites, while claudins -3 and -4 appear until gestational day 7 and only at implantation sites [44], reinforcing the view of claudin-4 as an important TJ proteins for embryonic development. Moreover, in rat uterus, by the time of implantation of the blastocyst at gestational day 6, when the network of TJ strands increases 3-fold in depth along the lateral plasma membrane and displays more branches and interconnections with neighboring strands [45,46], claudin-4 is detected for the first time at the basolateral membrane of uterine epithelial cells [44]. Similarly, in human endometrium, an increase in claudin-4 mRNA is found during the implantation window [47,48,49], thus suggesting a critical role of claudin-4 during implantation.

The role of claudin-4 in human placenta is highlighted by the observation that its expression increases in hydatidiform moles and in maternal diabetes [11]. In human placentae derived from assisted reproductive technology, claudin-4 mRNA diminishes, and this change is accompanied by an increase in claudin-8 mRNA [50]; while in mice placentae, claudin-4 augments as gestation advances from days 12 to 20 and after the administration of the estrogen receptor antagonist ICI 182,780 and the progesterone receptor antagonist RU-486 [36].

How ZIKV alters the Cl-4 expression of STB without actively replicating in these cells remains an open question. It has been observed that a wide array of viruses use integral proteins located at the apical junctional complex (AJC) of epithelial cells (for review see [51]) as cellular receptors, including for example hepatitis C virus, that associates to claudins -1, -6, and -9 [52,53]. The use of such proteins is important for the entry of viruses into epithelial cells, but also implies the disruption of the AJC that compromises the integrity of the epithelial barrier in consequence. Thus, West Nile virus specifically induces the endocytosis of claudin-1 and JAM-A [54], and in rotavirus, the VP8 protein, generated from the proteolytic cleavage of spike protein VP4, opens the TJs of epithelial cells in a reversible and dose-dependent manner [55]. VP8 has several segments with high similarity to domains present in the extracellular loops of claudins and occludin; hence, it was proposed that VP8 opened the TJs by competing with the homotypic interactions established among the extracellular domains of certain claudins and occludin. Another interesting protein in this respect, derived from a microorganism, is *Clostridium perfringens* enterotoxin (CPE). This toxin opens the paracellular barrier due to the selective removal of claudins -4 and -3 from the TJ [56,57]. In the case of ZIKV, the effect of structural E and M proteins on TJ proteins has not yet been explored.

The observation that only the basolateral exposure of ZIKV reduced TER and claudin-4 expression of BeWo monolayers is not surprising, as a similar situation had been observed with several viruses. Thus, the adenovirus fiber protein can only access its CAR receptor at the apical junctional complex and open the TJs when added to the basolateral surface [20]; HSV-1 only infected epithelial cells if added to the basolateral surface or if depletion of extracellular calcium had weakened the strength of the AJC to allow the virus to access its nectin receptor [58,59]; and hepatitis C virus first localizes with the epidermal growth factor receptor at the basolateral membrane and then accumulates at the TJ and associates to claudin-1 and occludin [60,61]. These results suggest that ZIKV passed from the maternal basal decidua to the fetal invading CTB and the cell columns of CTB, or through the transport system facilitated by the neonatal Fc receptor to transcytose across the STB layer, could open the paracellular pathway of the STB layer due to its presence in the parenchyma of chorionic villi that faces the basolateral surface of STB cells. Hence, the opening of the TJ in the STB could occur not as an initial step in the vertical transmission of ZIKV, but as a consequence of chorionic villi infection.

The heterogeneous maturation of chorionic villi, Hofbauer cell hyperplasia, and intravillous calcifications that we observed in ZIKV-infected placentae have also been reported in other studies [15,62,63,64]. In this respect, alterations in Hofbauer cells homeostasis are known to be associated with placental pathologies involving infection, inflammation, and inadequate placental development [65]. With regards to the diameter of chorionic villi, as the third trimester of pregnancy advances, stem villi branch into distal villi; in consequence, the diameter of chorionic villi decreases [66]. Thus, the higher diameter of chorionic villi observed in placentae from ZIKV-infected women suggests villous maldevelopment, although an effect due to the different ages of ZIKV and control placenta cannot be disregarded. Nevertheless, it should also be mentioned that this effect could be related to the altered expression of claudin-4, since in both mice and humans, during placental development, frizzled 5 induces the disassociation of cell junctions for chorion branching initiation through the downregulation of ZO-1, claudin-4 and claudin-7 in trophoblast cells [67]. Therefore, another important aspect to study in the future in ZIKV-infected placentae could be the expression of frizzled 5.

**Figure 6:**
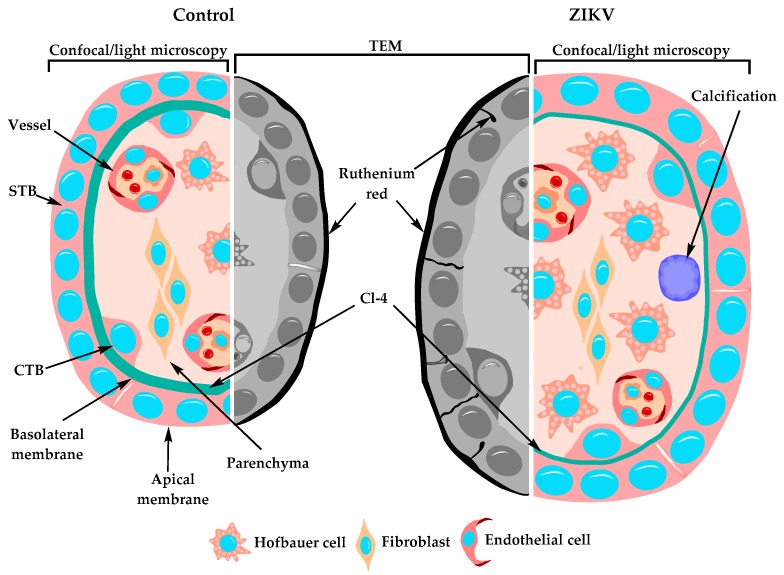
Schematic representation of the changes observed in chorionic villi of ZIKV-infected women. Chorionic villi derived from women infected with ZIKV during pregnancy, displayed several alterations including Hofbauer cell hyperplasia, increased diameter of microvilli, intravillous calcifications, and a STB layer with a diminished expression of claudin-4 and permeable to ruthenium red passage though the paracellular pathway.

In summary, our results indicate that the chorionic villi of placentae from women infected with ZIKV display Hofbauer cell hyperplasia, an increased diameter of microvilli and intravillous calcifications, while the study of the STB layer of these placentae shows a decreased expression of claudin-4 and ruthenium red permeability, suggesting that these placentae are leakier than the normal, control ones (Figure 6). These observations allowed us to propose the paracellular pathway of the STB layer as a route of vertical transmission of ZIKV. However, the observation that ZIKV only reduced the TER of a trophoblast cell line when added to the basolateral surface raises the possibility of seeing the opening of TJs in the STB as a consequence of ZIKV infection of the chorionic villi.

## Figures and Tables

**Table 1:** Clinical data of women with ZIKV during pregnancy and physical findings of their newborns.

**Mother**					
**Donor**	**Age (years)**	**Birth (GW)**	**Zika Symptoms Onset (GW)**	**Rash**	**Pregnancy Complications**
D1	31	36.6	11.5	Yes	Preeclampsia
D5	35	35.1	7.6	Yes	Preeclampsia
D8	30	36.0	13.0	Yes	None
D10	29	36.0	15.0	Yes	None
**Newborn**					
**Sex**	**Weight (kg)**	**Height (cm)**	**Apgar**	**HC (cm)**	**Outcome**
F	2.84	47	8/9	33.0	Right pulmonary cystic adenomatoid malformation
F	2.92	47	6/9	36.0	Ventriculomegaly, macrocephaly, hydrocephaly, and hip dysplasia
F	3.00	48	8/9	33.8	22q11 deletion syndrome with mielomeningocele, hypotonia and right aortic arch
F	3.03	50	8/9	34.0	Brachycephaly, low-set ears, short neck, and widely-spaced nipples

GW, Gestational week; HC, Head circumference; F, Female.

**Table 2:** Clinical data of control women during pregnancy and physical findings of their newborns.

**Mother**									
Donor		Age (years)		Birth (GW)			Pregnancy complications		
D9		32		38.0			Preeclampsia		
D17		30		38.8			Preeclampsia		
D18		23		40.0			None		
D19		34		36.0			None		
**Newborn**									
Sex	Weight (kg)		Height (cm)		Apgar	HC (cm)		Outcome	
M	2.20		50		8/9	35.0		Healthy	
F	2.90		49		8/9	34.0		Healthy	
M	3.20		51		8/9	36.0		Healthy	
F	2.97		50		9/9	35.0		Healthy	

GW, Gestational week; F, Female; M, Male; HC, Head circumference.

**Table 3:** Expression of E-cadherin and TJ proteins in the trophoblast of placental chorionic villi derived from control and ZIKV-infected women.

Protein	STB Distribution	Abundance in Chorionic Vessels	ZIKV-infected vs. Control Placentae
E-cadh	basal	**−**	**=**
Cl-1	apical/basal	**+++**	**=**
Cl-2	nd	**−**	
Cl-3	apical/basal	**+++**	**=**
Cl-4	basal	**+++**	**↓**
Cl-5	apical/basal	**+++**	**=**
Cl-7	basal	**±**	**=**
Cl-10	nd	**−**	
JAM-A	nd	**−**	
JAM-B	apical/basal	**−**	**=**
JAM-C	bd	**+++**	**=**
Occ	apical/basal	**+++**	**=**
ZO-1	apical/basal	**+++**	**=**
ZO-2	apical/basal	**±**	**=**

BD, barely detected; ND, not detected. −, absent; ±, barely detected; +++ highly abundant; =, no change; ↓, decrease.

## Supplementary Materials

The following are available online at https://www.mdpi.com/2073-4409/8/10/1174/s1, Figure S1. E-cadherin presence in the basolateral membrane of the STB was not altered in placental tissue from ZIKV-infected women, Figure S2. The expression of claudin-1 in the STB of chorionic villi in human placenta increased with ZIKV infection, Figure S3: Claudin-3 was strongly expressed in vessels in the chorionic parenchyma, Figure S4. Claudin-5 was strongly expressed in chorionic villi vessels and faintly stained the STB cell layer, Figure S5: Claudin-7 is present in the basolateral surface of STB cells and infection with ZIKV did not alter its expression, Figure S6. ZO-1 strongly stains the chorionic vessels, Figure S7. ZO-2 stained the STB layer of placentae, Figure S8. JAM-B was present in the STB cell layer and its expression was not affected by ZIKV infection, Figure S9: JAM-C expression was abundant in chorionic vessels but scarce in the STB layer, Figure S10: Chorionic villi stained with haematoxylin and eosin in D1 placenta from a ZIKV-infected woman, Figure S11: Chorionic villi stained with haematoxylin and eosin in D5 placenta from a ZIKV-infected woman, Figure S12: Chorionic villi stained with haematoxylin and eosin in D8 placenta from a ZIKV-infected woman, Figure S13: Chorionic villi stained with haematoxylin and eosin in D10 placenta from a ZIKV-infected woman, Figure S14: Chorionic villi stained with haematoxylin and eosin in D9 placenta from the control group, Figure S15: Chorionic villi stained with haematoxylin and eosin in placenta D17 from the control group, Figure S16: Chorionic villi stained with haematoxylin and eosin in placenta D18 from the control group, Figure S17: Chorionic villi stained with haematoxylin and eosin in placenta D19 from the control group, Figure S18: Chorionic villi stained with Masson’s trichrome stain in D1 placenta from a ZIKV-infected woman.
