# Supplementary material for: Syncytiotrophoblast of Placentae from Women with Zika Virus Infection Has Altered Tight Junction Protein Expression and Increased Paracellular Permeability
Source: Cells. 2019 Sep 29;8(10):1174. doi: 10.3390/cells8101174 (PMC6829373; doi:10.3390/cells8101174)
Supplement: Supplementary file 1 [file cells-08-01174-s001.pdf]

Supplementary Materials

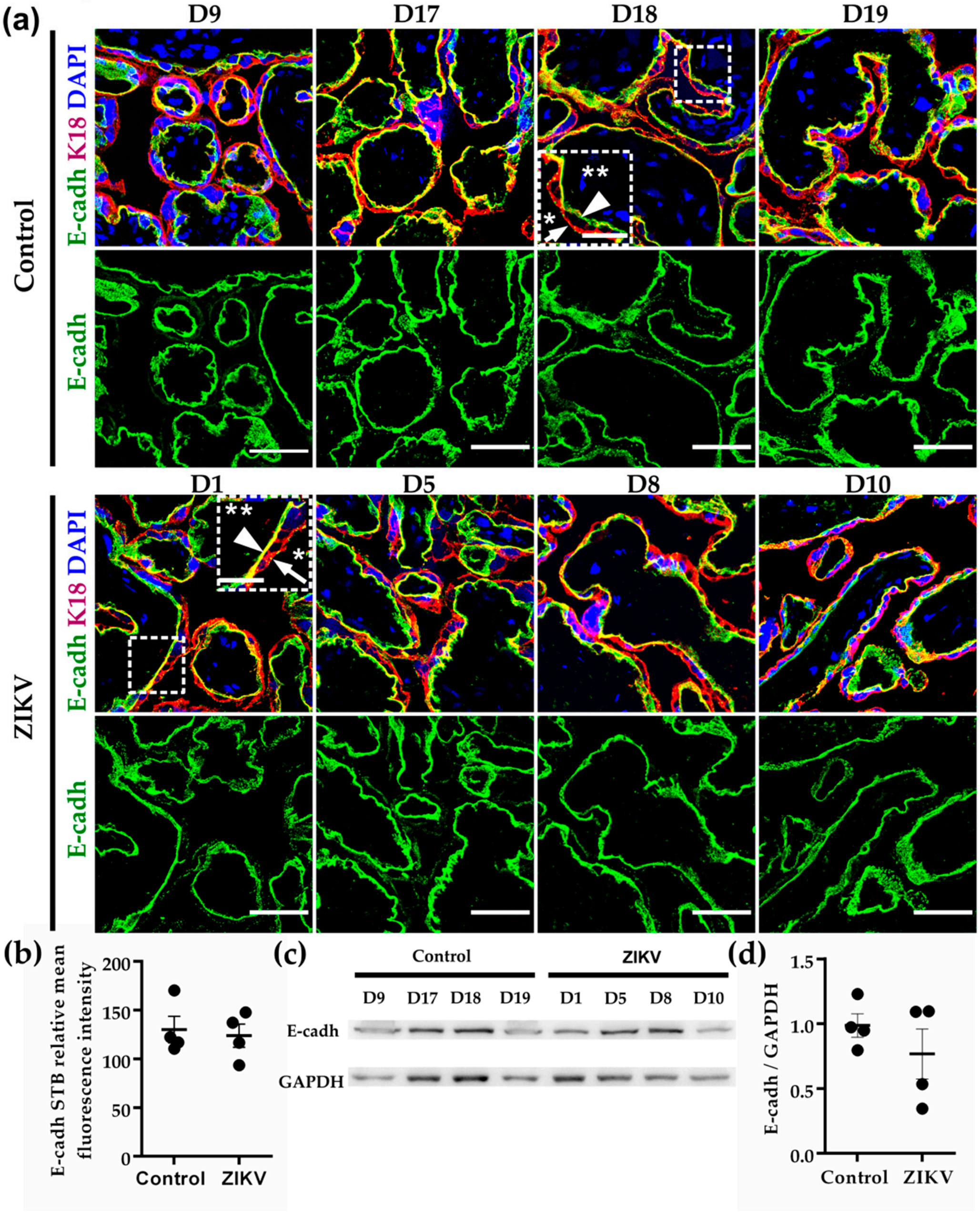

Figure S1. E-cadherin presence in the basolateral membrane of the STB was not altered in placental tissue from ZIKV infected women. (a) Frozen sections of human placentae derived from women infected or not (control) with ZIKV were processed for immunofluorescence with a rabbit antibody against E-cadherin (E-cadh) and a mouse antibody anti cytokeratin 18 (K18). DNA of nuclei was stained with DAPI. Apical membrane of STB cells in contact with the intervillous space, stained only with K18 (arrows); basolateral membrane of STB cells in contact with the chorionic villi parenchyma stained with both K18 and E-cadherin (arrowheads); intervillous space (asterisk); chorionic parenchyma (double asterisk). Bar, 50 µm; magnification bar, 25 µm. (b) Measurements of mean fluorescence intensity of the trophoblast layer were done on three independent images from each condition. (c) Representative Western blot of three independent experiments. Glyceraldehyde-3-phosphate dehydrogenase (GAPDH) was employed as loading control. (d) Densitometric analysis of Western blots.

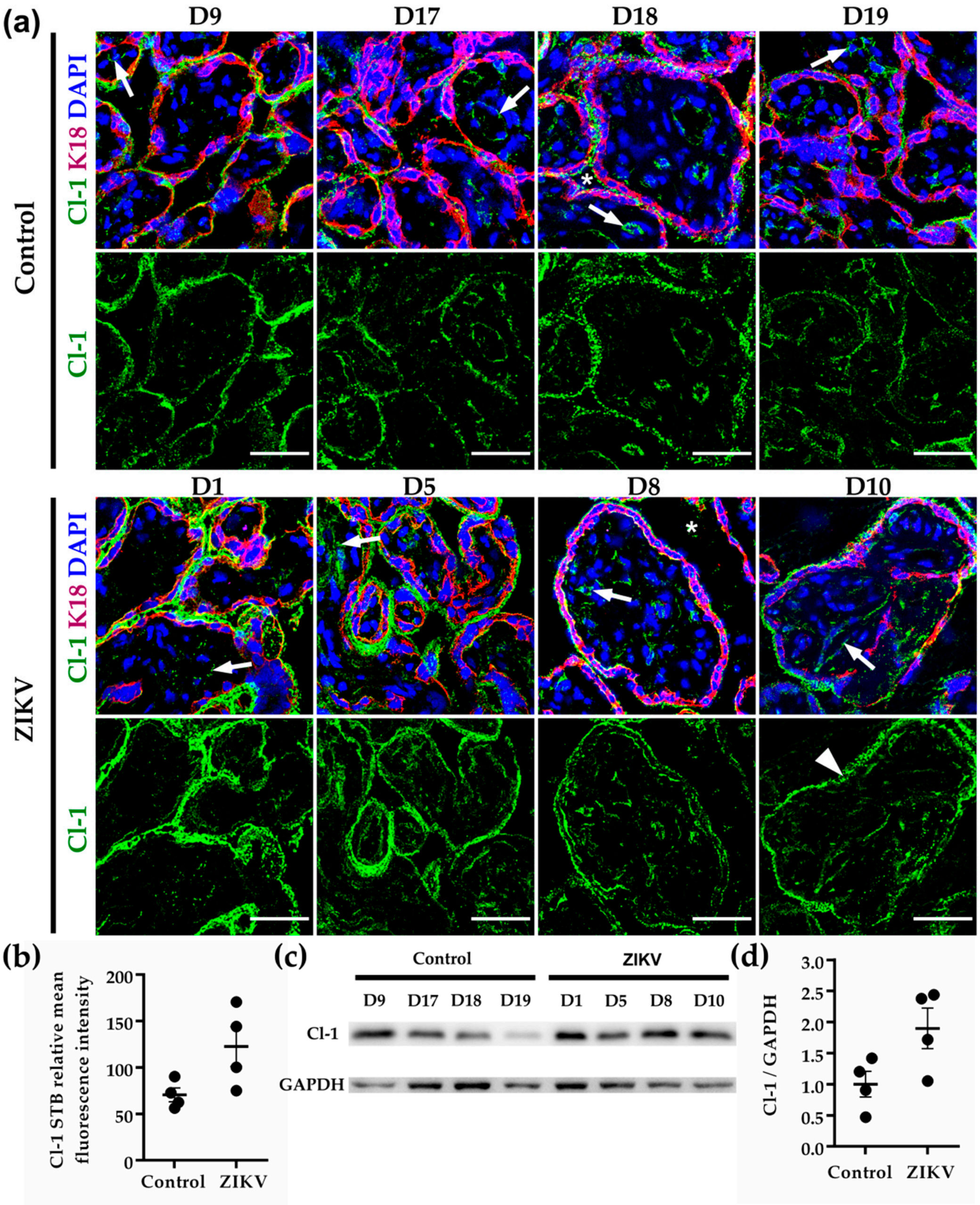

Figure S2. The expression of claudin-1 in the STB of chorionic villi in human placenta increased with ZIKV infection. (a) Frozen sections of human placentae derived from women infected or not (control) with ZIKV were processed for immunofluorescence with a rabbit antibody against claudin-1 (Cl-1) and a mouse antibody anti cytokeratin 18 (K18). DNA of nuclei was stained with DAPI. Chorionic vessels (arrow); STB layer (arrowhead); intervillous space (asterisks). Bar, 50 µm. (b) Measurements of mean fluorescence intensity of the trophoblast layer were done on three independent images from each condition. (c) Representative Western blot of three independent experiments. GAPDH was employed as loading control. (d) Densitometric analysis of Western blots.

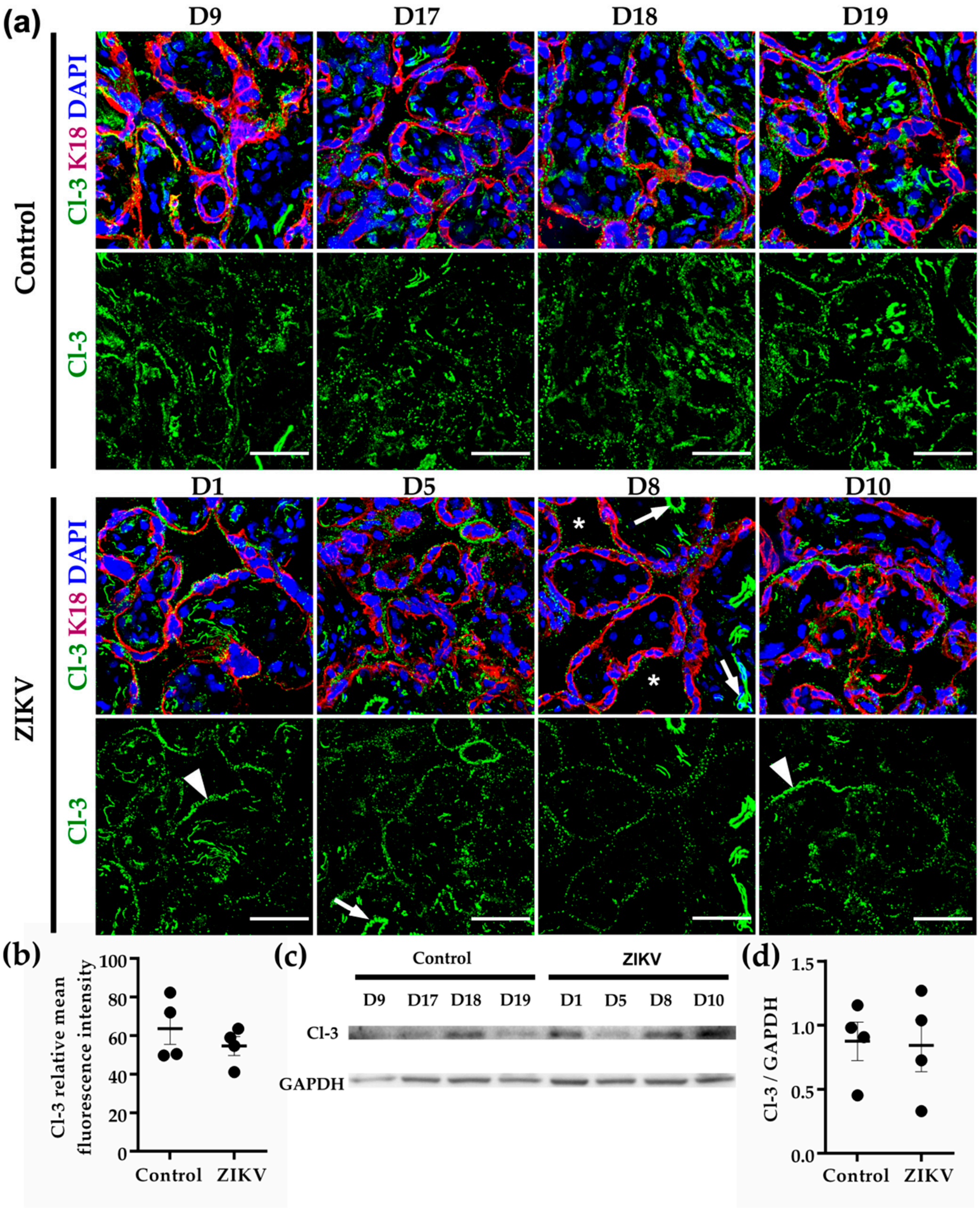

Figure S3. Claudin-3 was strongly expressed in vessels in the chorionic parenchyma. (a) Frozen sections of human placentae derived from women infected or not (control) with ZIKV were processed for immunofluorescence with a rabbit antibody against claudin-3 (Cl-3) and a mouse antibody anti cytokeratin 18 (K18). DNA of nuclei was stained with DAPI. Vessels in the chorionic parenchyma (arrows); STB cell layer (arrowheads); intervillous space (asterisks). Bar, 50 m. (b) Measurements of mean fluorescence intensity of trophoblast layer were done on three independent images from each condition. (c) Representative Western blot of three independent experiments. GAPDH was employed as loading control. (d) Densitometric analysis of Western blots.

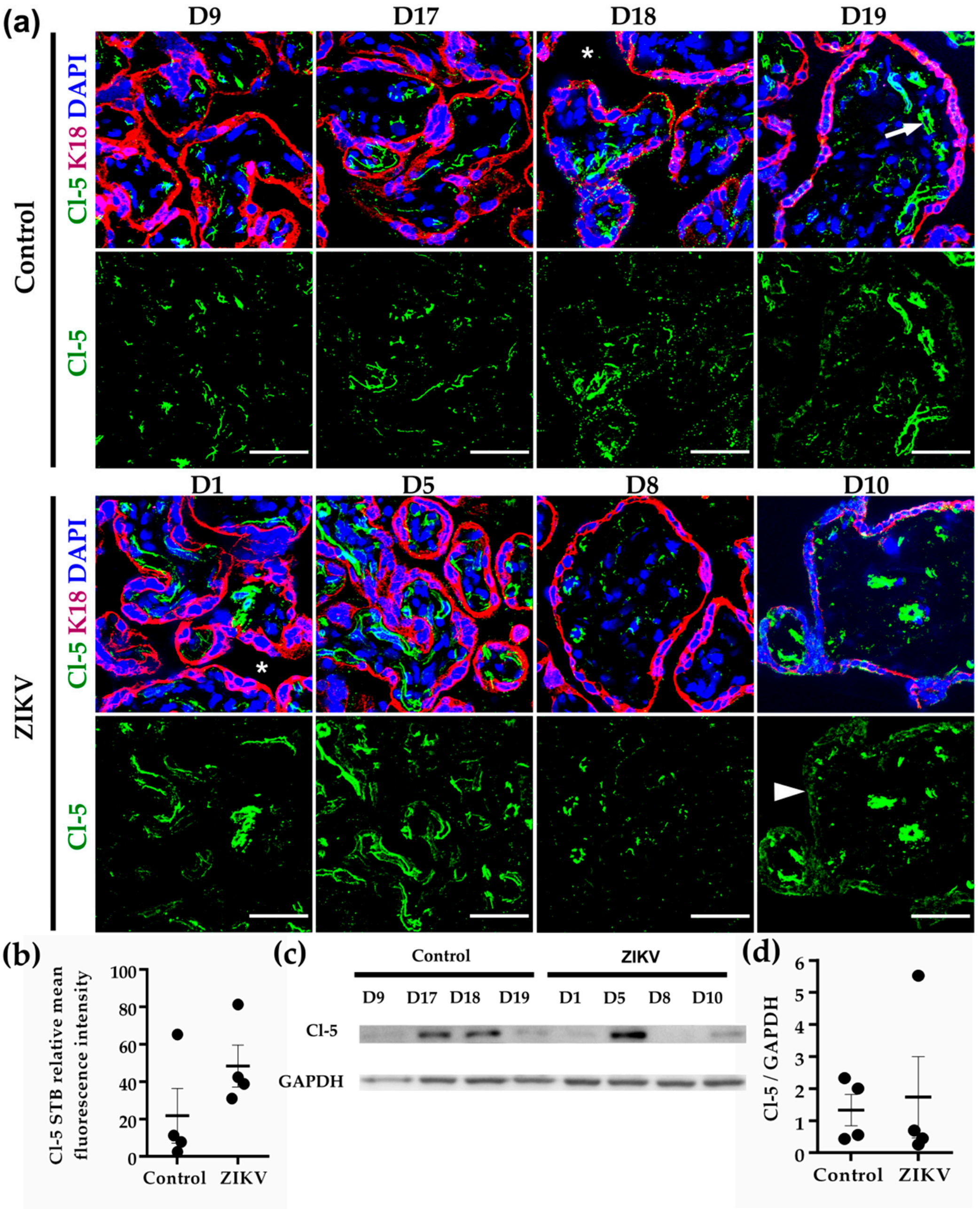

Figure S4. Claudin-5 was strongly expressed in chorionic villi vessels and faintly stained the STB cell layer. (a) Frozen sections of human placentae derived from women infected or not (control) with ZIKV were processed for immunofluorescence with a rabbit antibody against claudin-5 (Cl-5) and a mouse antibody anti cytokeratin 18 (K18). DNA of nuclei was stained with DAPI. Bar, 50 μm. Chorionic vessels (arrow); STB cell layer (arrowhead); intervillous space (asterisk). (b) Measurements of mean fluorescence intensity of the trophoblast layer were done on three independent images from each condition. (c) Representative Western blot of three independent experiments. GAPDH was employed as loading control. (d) Densitometric analysis of Western blots.

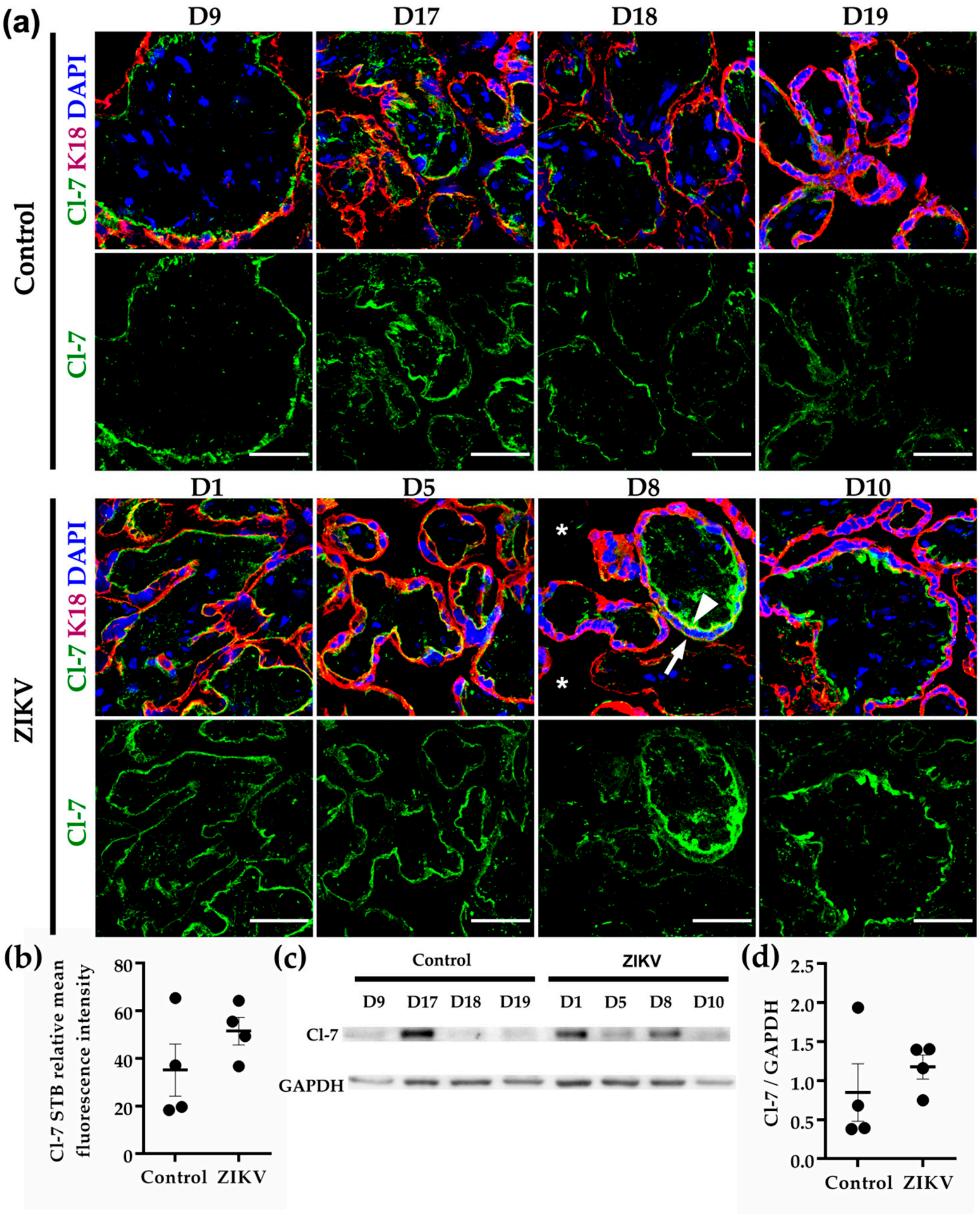

Figure S5. Claudin-7 is present in the basolateral surface of STB cells and infection with ZIKV did not alter its expression. (a) Frozen sections of human placentae derived from women infected or not (control) with ZIKV were processed for immunofluorescence with a rabbit antibody against claudin-7 (Cl-7) and a mouse antibody anti cytokeratin 18 (K18). DNA of nuclei was stained with DAPI. Apical membrane of STB cells (arrow); basolateral membrane of STB cells (arrowhead); intervillous space (asterisks). Bar, 50 µm. (b) Measurements of mean fluorescence intensity of trophoblast layer were done on three independent images from each condition. (b) Left, representative Western blot of three independent experiments. GAPDH was employed as loading control. (d) Densitometric analysis of Western blots.

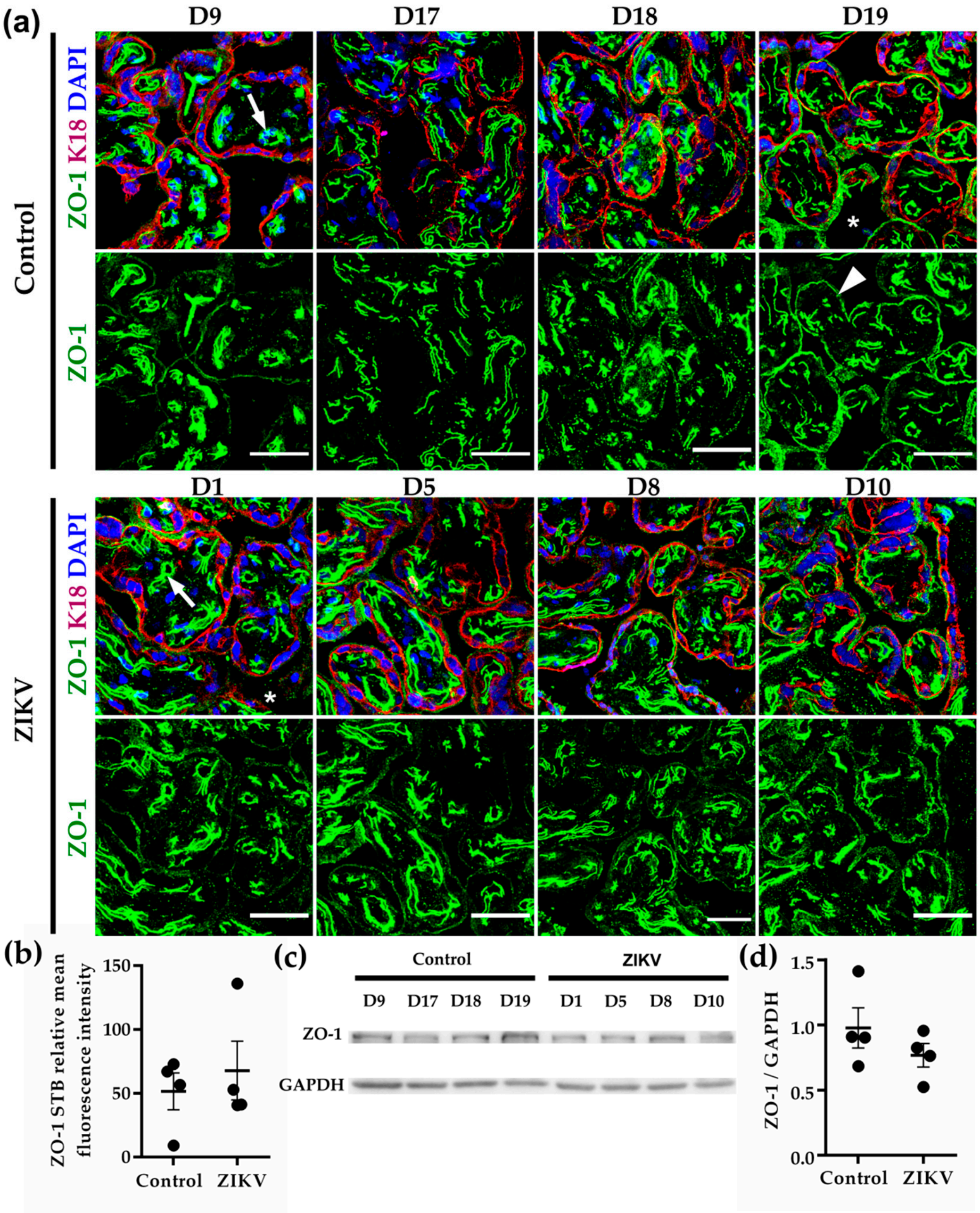

Figure S6. ZO-1 strongly stains the chorionic vessels. (a) Frozen sections of human placentae derived from women infected or not (control) with ZIKV were processed for immunofluorescence with a rabbit antibody against ZO-1 and a mouse antibody anti cytokeratin 18 (K18). DNA of nuclei was stained with DAPI. Fetal endothelia within the chorionic villi parenchyma (arrows); STB (arrowhead); intervillous space (asterisk). Bar, 50 μm. (b) Measurements of mean fluorescence intensity of the trophoblast layer were done on three independent images from each condition. (c) Representative Western blot of four independent experiments. GAPDH was employed as loading control. (d) Densitometric analysis of Western blots.

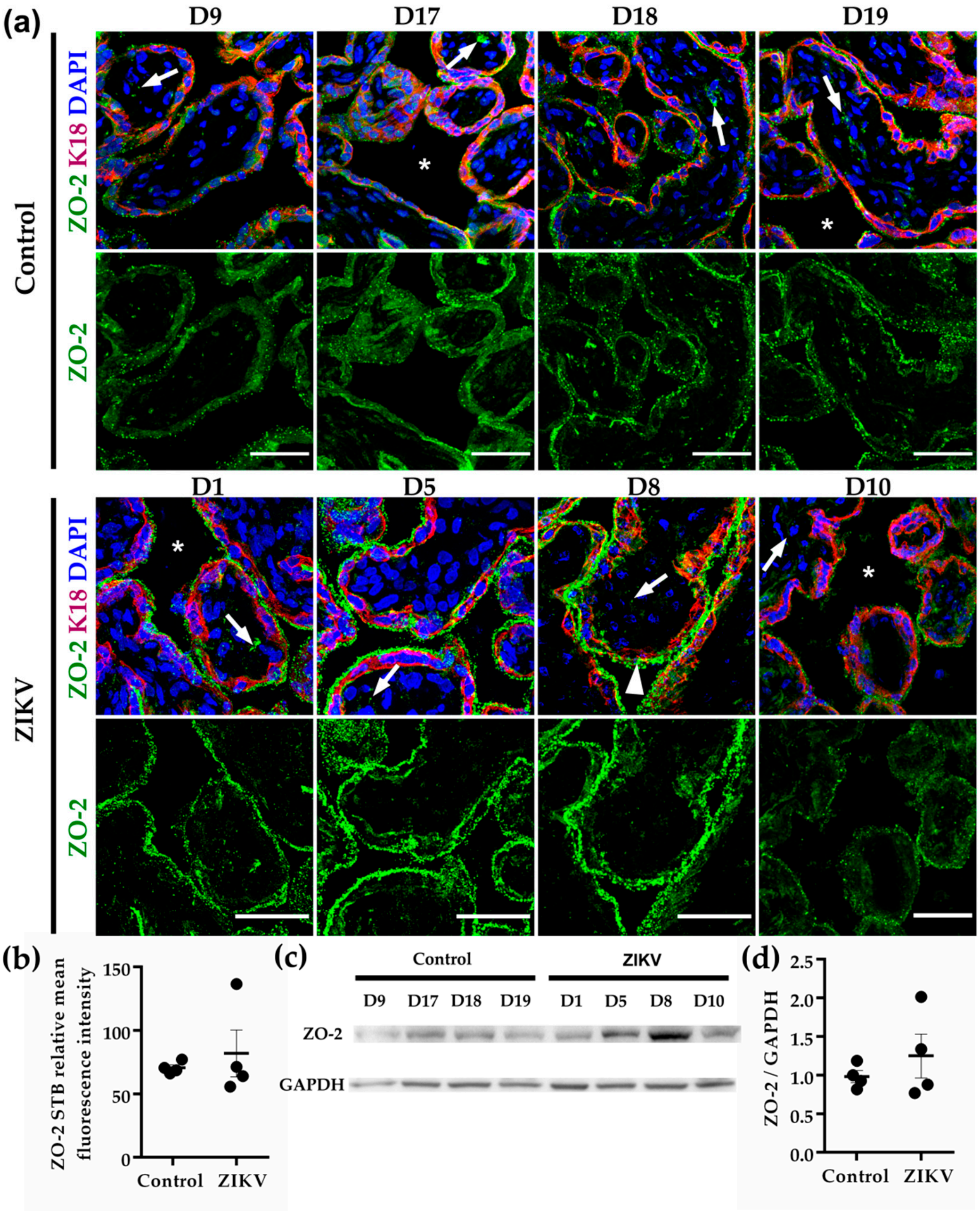

Figure S7. ZO-2 stained the STB layer of placentae. (a) Frozen sections of human placentae derived from women infected or not (control) with ZIKV were processed for immunofluorescence with a rabbit antibody against ZO-2 and a mouse antibody anti cytokeratin 18 (K18). DNA of nuclei was stained with DAPI. Fetal endothelia within the chorionic villi parenchyma (arrows); STB (arrowhead); intervillous space (asterisks). Bar, 50 µm. (b) Measurements of mean fluorescence intensity of the trophoblast layer were done on three independent images from each condition. (c) Representative Western blot of four independent experiments. GAPDH was employed as loading control. (d) Densitometric analysis of Western blots.

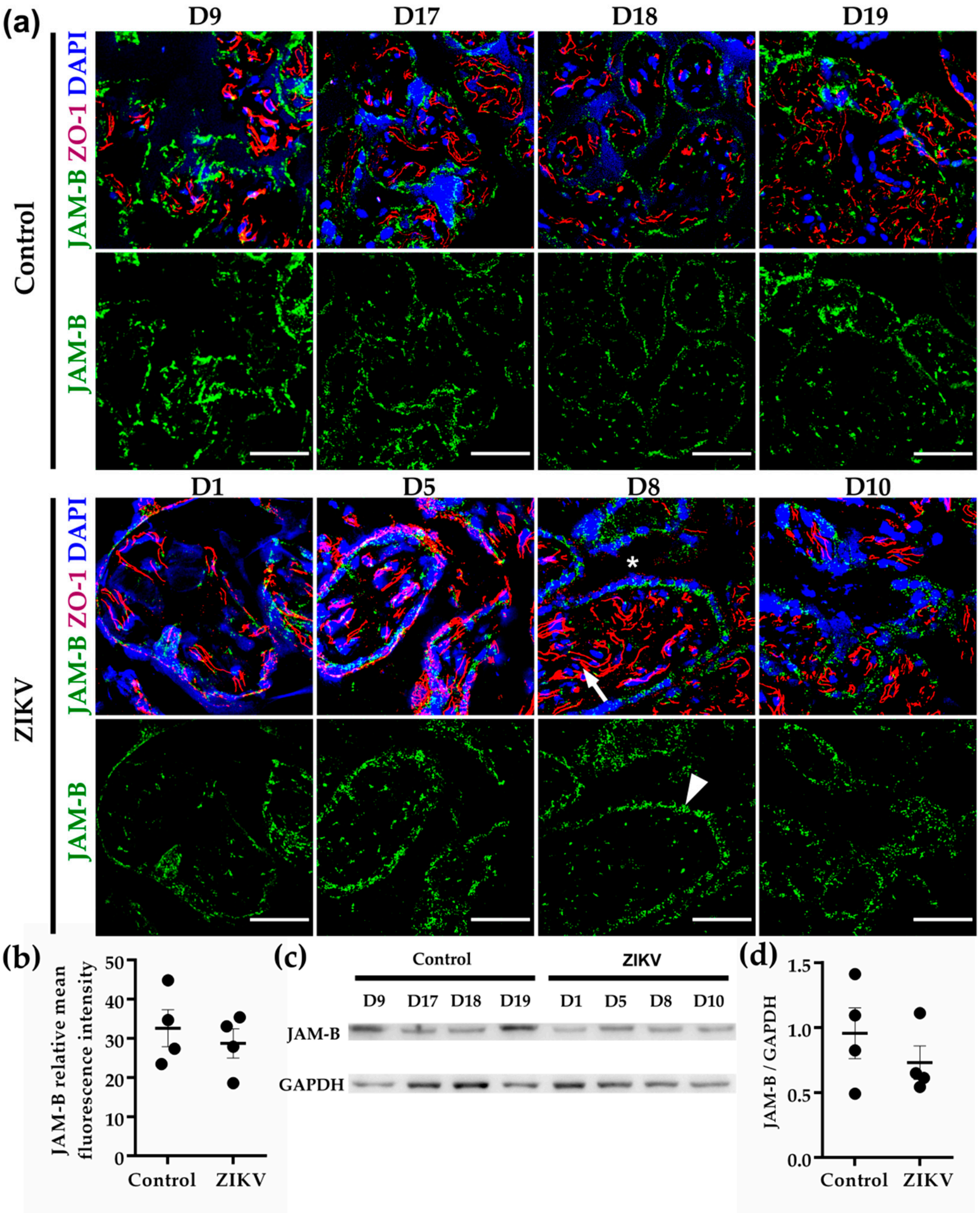

Figure S8. JAM-B was present in the STB cell layer and its expression was not affected by ZIKV infection. (a) Frozen sections of human placentae derived from women infected or not (control) with ZIKV were processed for immunofluorescence with a rabbit antibody against ZO-1 and a mouse antibody anti JAM-B. DNA of nuclei was stained with DAPI. Fetal endothelia within the chorionic villi parenchyma (arrow); STB (arrowhead); intervillous space (asterisk). Bar, 50 µm. (b) Measurements of mean fluorescence intensity of the trophoblast layer were done on three independent images from each condition. (c) Representative Western blot of three independent experiments. GAPDH was employed as loading control. (d) Densitometric analysis of Western blots.

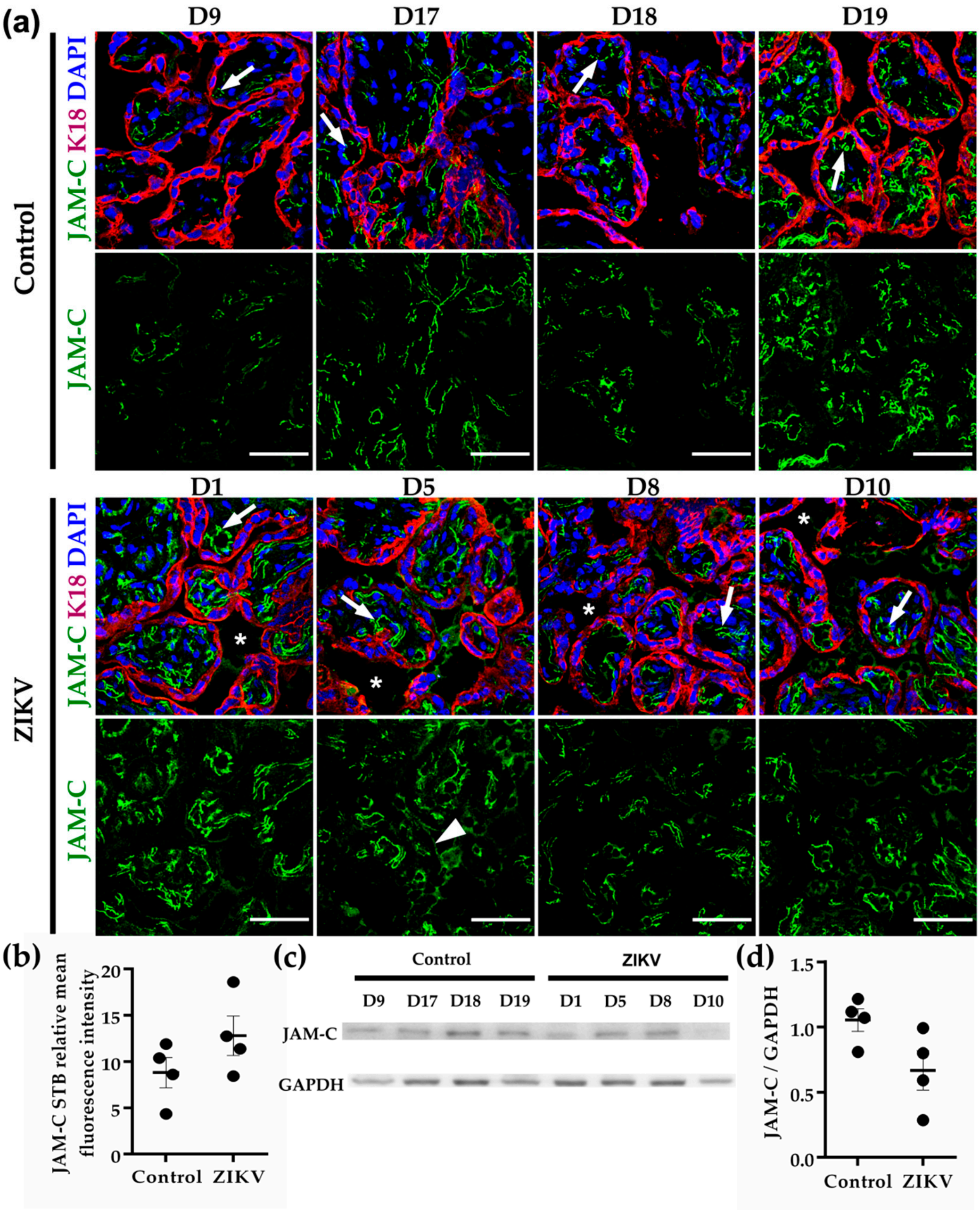

Figure S9. JAM-C expression was abundant in chorionic vessels but scarce in the STB layer. (a) Frozen sections of human placentae derived from women infected or not (control) with ZIKV were processed for immunofluorescence with a rat antibody anti JAM-C and a mouse antibody anti cytokeratin 18 (K18). Nuclei were stained with DAPI. Fetal endothelia within the chorionic villous parenchyma (arrows); STB cell layer (arrowheads); intervillous space (asterisks). Bar, 50 µm. (b) Measurements of mean fluorescence intensity of trhophoblast layer were done on three independent images from each condition. (c) Representative Western blot of three independent experiments. GAPDH was employed as loading control. (d) Densitometric analysis.

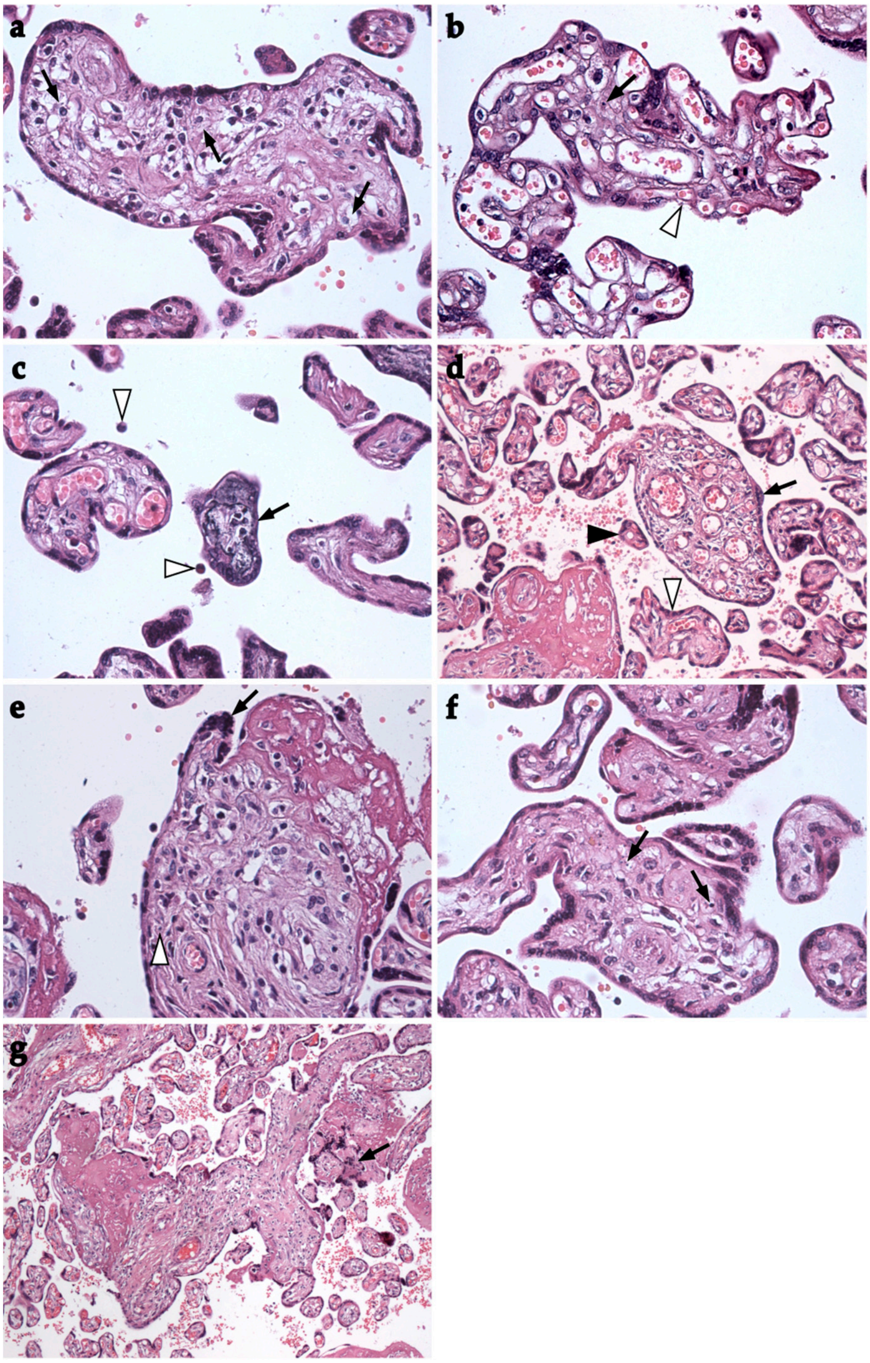

Figure S10. Chorionic villi stained with haematoxylin and eosin in D1 placenta from a ZIKV infected woman. (a) Hofbauer hyperplasia. Hofbauer cells (arrows), 40X. (b) Chorionic villi edema (arrow) and loss of syncytiotrophoblast (arrowhead), 40X. (c) Intravillous calcification (arrow) and eosinophils (arrowheads), 40X. (d) Heterogeneous maturation of chorionic villi. Immature intermediate villi (arrow) co-exist with secondary (empty arrowhead) and tertiary villi (full arrowhead), 10X. (e) Increased syncytial knots due to premature aging (arrow) and karyorrhexis (arrowhead), 40X. (f) Karyorrhexis in a chorionic villus (arrows), 40X. (g) Lymphocyte infiltration (arrow) denotes inflammation of chorionic villi, 40X.

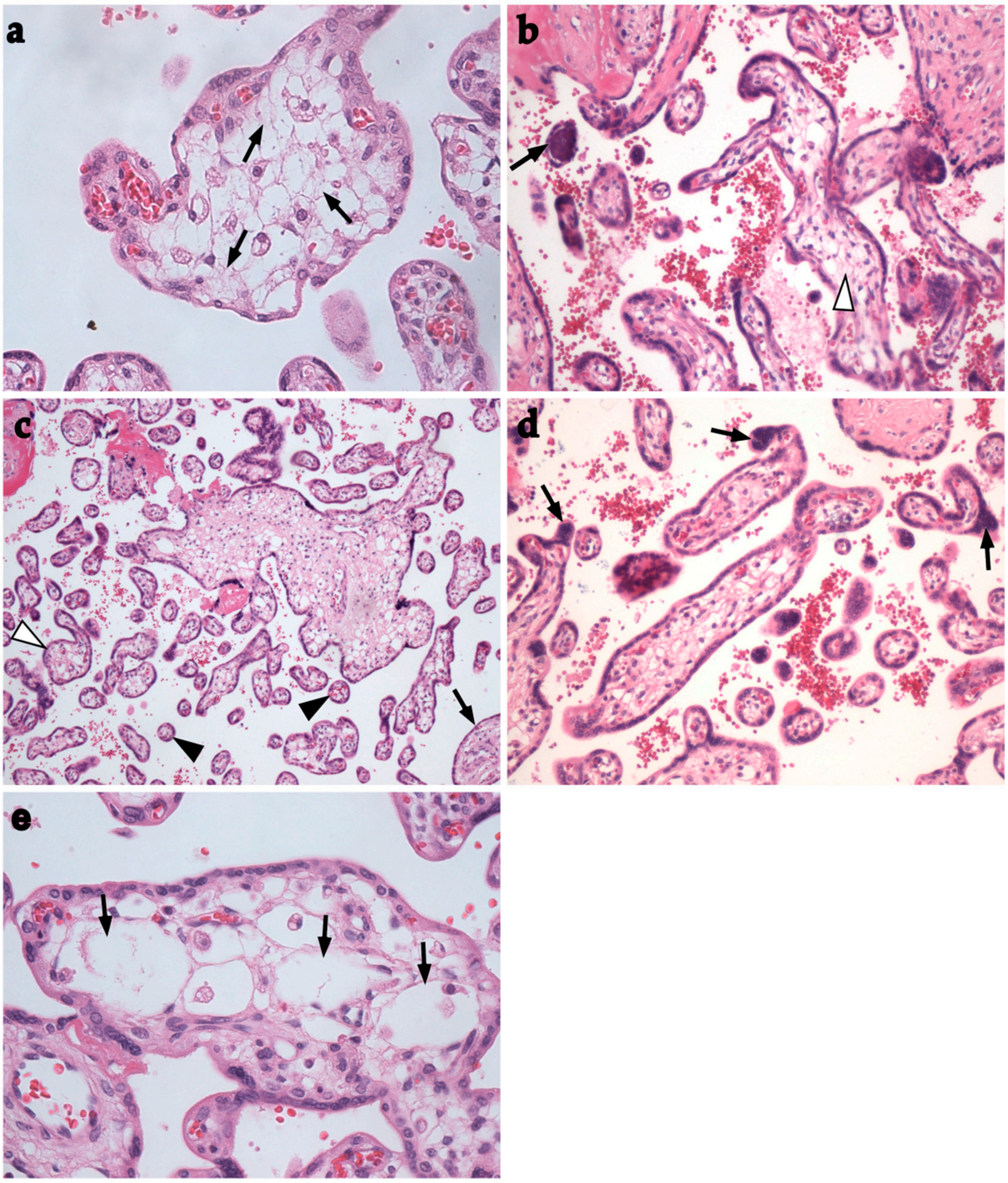

Figure S11. Chorionic villi stained with haematoxylin and eosin in D5 placenta from a ZIKV infected woman. (a) Chorionic villi edema (arrows), 40X. (b) Intravillous calcification (arrow) and chorionic villi edema (arrowhead), 20X. (c) Heterogeneous maturation of chorionic villi. Immature intermediate villi (arrow) co-exist with secondary (empty arrowheads) and tertiary villi (full arrowhead), 10X. (d) Increased syncytial knots (arrows) due to premature aging, 20X. (e) Intravillous cysts (arrows), 40X.

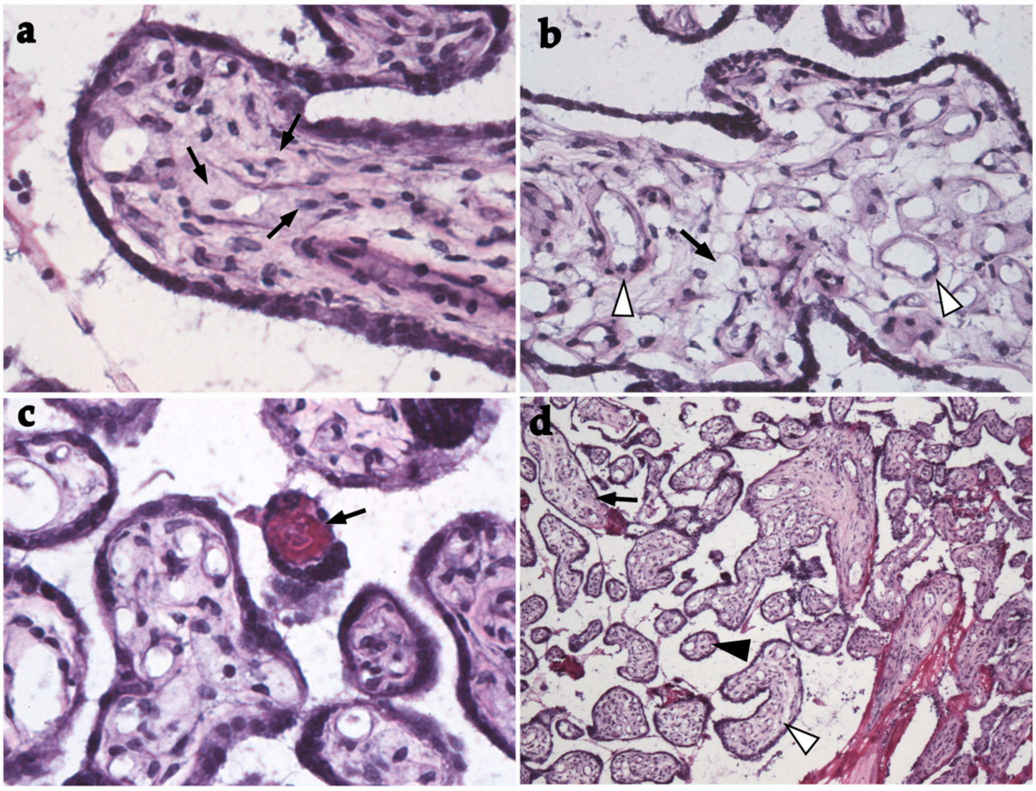

Figure S12. Chorionic villi stained with haematoxylin and eosin in D8 placenta from a ZIKV infected woman. (a) Hofbauer cell hyperplasia. Hofbauer cells (arrows), 60X. (b) Chorionic villi edema (arrow) and vascular proliferation (arrowhead), 40X. (c) Intravillous hemorrhage (arrow). Maternal erythrocytes inside the chorionic villus, 60X. (d) Heterogeneous maturation of chorionic villi. Immature intermediate villi (arrow) co-exist with secondary (empty arrowhead) and tertiary villi (full arrowhead), 10X.

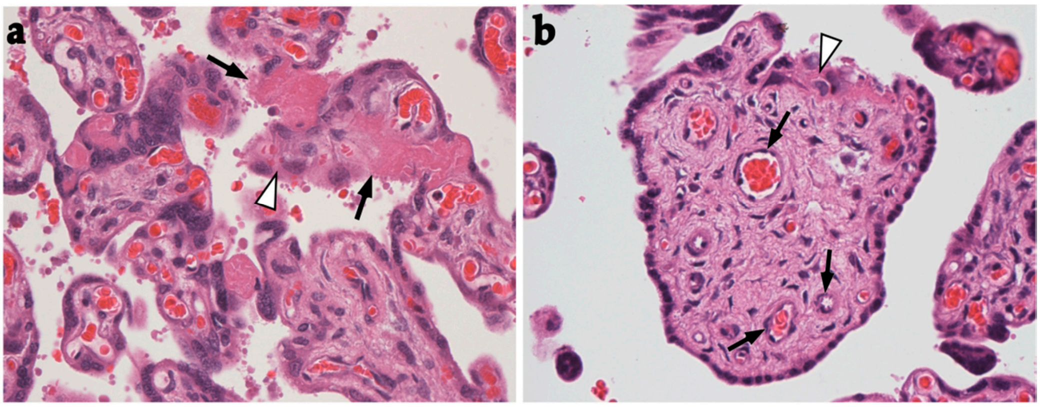

Figure S13. Chorionic villi stained with haematoxylin and eosin in D10 placenta from a ZIKV infected woman. (a) Abnormal appearance of fibrinogen (arrow), and loss of STB (arrowhead), 40X. (b) Vascular proliferation (arrows), and normal appearance of fibrinogen (arrowhead), 60X.

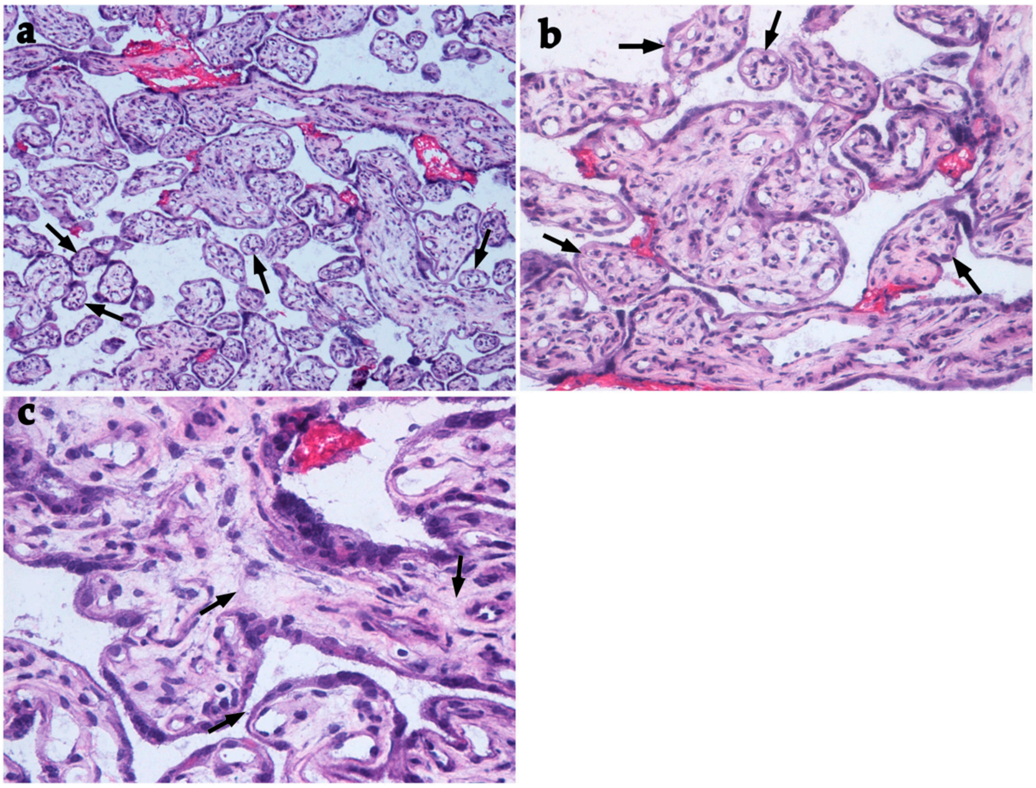

Figure S14. Chorionic villi stained with haematoxylin and eosin in D9 placenta from the control group. (a) Homogeneous maturation of chorionic villi. Tertiary villi (arrows), 10X. (b) Homogeneous maturation of chorionic villi. Tertiary villi (arrows), 20X. (c) Absence of parenchymal edema (arrows), 40X.

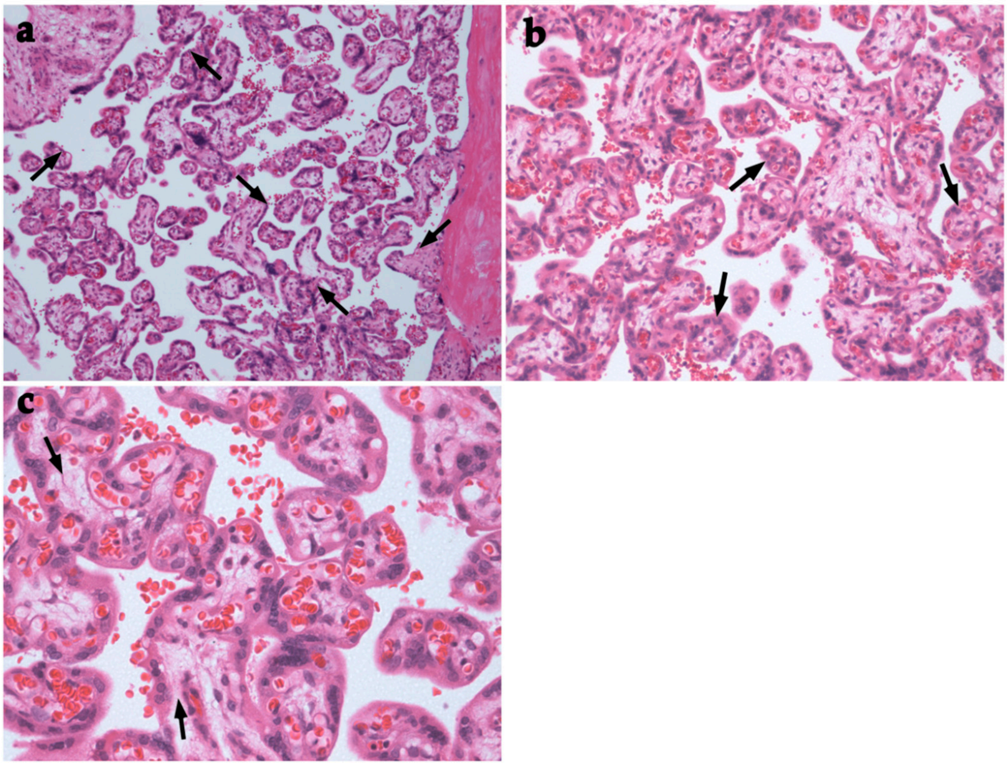

Figure S15. Chorionic villi stained with haematoxylin and eosin in placenta D17 from the control group. (a) Homogeneous maturation of chorionic villi. Tertiary villi (arows), 10X. (b) Homogeneous maturation of chorionic villi. Tertiary villi (arrows), 20X. (c) Absence of inflammatory cells. Microvilli parenchyma without segregated Hofbauer cells (arrows), 40X.

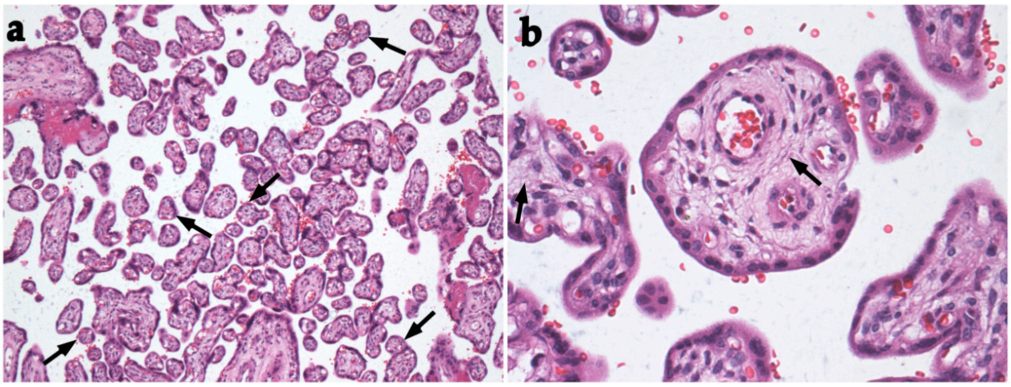

Figure S16. Chorionic villi stained with haematoxylin and eosin in placenta D18 from the control group. (a) Homogeneous maturation of chorionic villi. Tertiary villi (arrows), 10X. (b) Absence of inflammatory cells. Microvilli parenchyma without abundant Hofbauer cells (arrows), 40X.

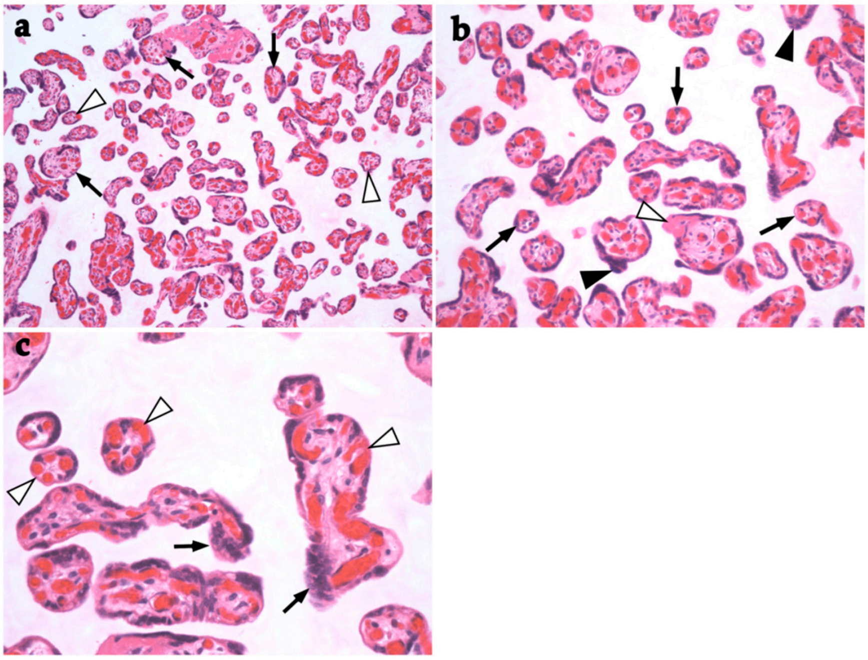

Figure S17. Chorionic villi stained with haematoxylin and eosin in placenta D19 from the control group. (a) Mature intermediate (arrows) and terminal chorionic villi (empty arrowhead), 10X. (b) Tertiary villi (arrow) normal appearance of fibrinogen (empty arrowhead), and syncitial knots (arrowheads), 20X. (c) Common syncytial knots (arrow), and vasculosyncitial membranes (empty arrowheads), 40X.

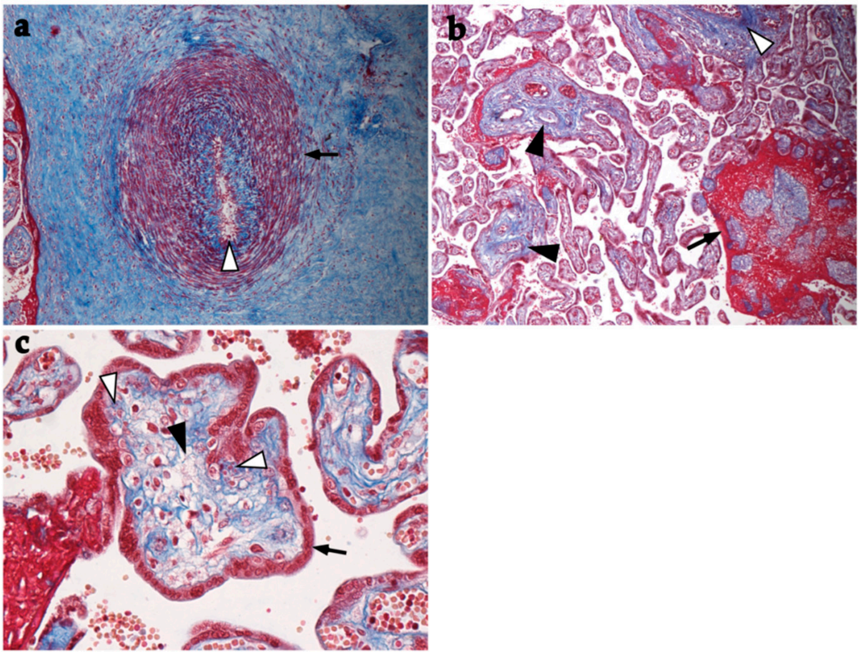

Figure S18. Chorionic villi stained with Masson's trichrome stain in D1 placenta from a ZIKV infected woman. (a) Decidual arterioles (arrow), vascular edema (arrowhead), 10X. (b) Excessive fibrinoid (arrow), perivascular fibosis (arrowhead) and abundant intravilli collagen (empty arrowheads), 10X. (c) Abnormally large microvillous (arrow), chorionic villi edema (arrowhead) and karyorrhexis (empty arrowheads), 40X.
